# Anterior gradient 2 induces resistance to sorafenib via endoplasmic reticulum stress regulation in hepatocellular carcinoma

**DOI:** 10.1186/s12935-023-02879-w

**Published:** 2023-03-10

**Authors:** Hung-Wen Tsai, Yi-Li Chen, Chun-I Wang, Ching‑Chuan Hsieh, Yang-Hsiang Lin, Pei-Ming Chu, Yuh-Harn Wu, Yi-Ching Huang, Cheng-Yi Chen

**Affiliations:** 1 Department of Pathology, National Cheng Kung University Hospital, College of Medicine, National Cheng Kung University, Tainan, Taiwan; 2 Department of Cell Biology and Anatomy, College of Medicine, National Cheng Kung University, Tainan, 70101 Taiwan; 3 Department of Biochemistry, School of Medicine, China Medical University, Taichung, Taiwan; 4 Division of General Surgery, Chang Gung Memorial Hospital, Chiayi, 613 Taiwan; 5 Liver Research Center, Chang Gung Memorial Hospital, Taoyuan, Taiwan; 6 Department of Anatomy, School of Medicine, Chung Shan Medical University, Taichung, Taiwan; 7 Department of Medical Education, Chung Shan Medical University Hospital, Taichung, Taiwan

**Keywords:** Hepatocellular carcinoma, Anterior gradient 2, Sorafenib, Resistance, Cancer progression, ER stress

## Abstract

**Background:**

Hepatocellular carcinoma (HCC) accounts for almost 80% of all liver cancer cases and is the sixth most common cancer and the second most common cause of cancer-related death worldwide. The survival rate of sorafenib-treated advanced HCC patients is still unsatisfactory. Unfortunately, no useful biomarkers have been verified to predict sorafenib efficacy in HCC.

**Results:**

We assessed a sorafenib resistance-related microarray dataset and found that anterior gradient 2 (AGR2) is highly associated with overall and recurrence-free survival and with several clinical parameters in HCC. However, the mechanisms underlying the role of AGR2 in sorafenib resistance and HCC progression remain unknown. We found that sorafenib induces AGR2 secretion via posttranslational modification and that AGR2 plays a critical role in sorafenib-regulated cell viability and endoplasmic reticulum (ER) stress and induces apoptosis in sorafenib-sensitive cells. In sorafenib-sensitive cells, sorafenib downregulates intracellular AGR2 and conversely induces AGR2 secretion, which suppresses its regulation of ER stress and cell survival. In contrast, AGR2 is highly intracellularly expressed in sorafenib-resistant cells, which supports ER homeostasis and cell survival. We suggest that AGR2 regulates ER stress to influence HCC progression and sorafenib resistance.

**Conclusions:**

This is the first study to report that AGR2 can modulate ER homeostasis via the IRE1α-XBP1 cascade to regulate HCC progression and sorafenib resistance. Elucidation of the predictive value of AGR2 and its molecular and cellular mechanisms in sorafenib resistance could provide additional options for HCC treatment.

**Supplementary Information:**

The online version contains supplementary material available at 10.1186/s12935-023-02879-w.

## Introduction

Hepatocellular carcinoma (HCC) is the most common hepatic malignant tumor and the 2^nd^ most common cause of cancer-related death worldwide [1]. Approximately 70% of patients are ineligible for curative therapy when they are diagnosed. Sorafenib is the first-line systemic therapy for advanced HCC to prolong survival [2]. Sorafenib is a multiple tyrosine kinase inhibitor (TKI) that inhibits numerous cell surface tyrosine kinases, such as vascular endothelial growth factor receptor (VEGFR)-1, VEGFR-2, VEGFR-3, platelet-derived growth factor receptor (PDGFR)-β and downstream associated serine/threonine kinases involved in the mitogen-activated protein kinase (MAPK) cascade [3, 4]. In vitro, sorafenib blocks cell proliferation and induces cell apoptosis in HCC cell lines, and in vivo*,* sorafenib inhibits tumor growth and induces tumor cell apoptosis [5]. Sorafenib is effective in increasing the median survival time of HCC patients by approximately 3–5 months. However, several side effects are associated with sorafenib treatment and are usually followed by drug resistance [6]. Several mechanisms and pathways related to acquired resistance to sorafenib have been identified; these include the epithelial-mesenchymal transition (EMT) mechanism and activation of hypoxia-inducible pathways, the phosphatidylinositol-3-kinase (PI3K)/Akt pathway, and the Janus kinase-signal transducer and activator of transcription (JAK-STAT) pathway [6]. Moreover, JAK/STAT pathway-related molecules, such as phospho-STAT3 and its downstream proapoptotic proteins Mcl-1 and cyclin D1, exhibit dysregulated expression in HCC cell lines with sorafenib resistance [7]. Previously, Liovet et al. reported that HCC progression is enhanced after sorafenib treatment through paracrine secretion of hepatocyte growth factor by stromal cells stimulated by VEGFA [8]. Unfortunately, thus far, there are no useful markers to predict the efficiency of sorafenib targeted therapy in HCC.

In the present study, we retrieved information from two databases: the sorafenib-resistant dataset for Huh7 cells by Regan-Fendt et al. (GSE94550, [9]) and the Roessler liver microarray dataset from Oncomine (GSE14520, [10]). To identify potential candidates for further study through intersection of the two datasets, we utilized a >  2-fold change as the criterion for the selection of sorafenib-modulated molecules in sorafenib-resistant Huh7 cells compared to parental cells (GSE94550) and a > 1.2-fold change as the criterion for choosing oncogenes related to survival in the bottom 25% vs. the top 25% of HCC patients from the Roessler Liver microarray (GSE14520). Following the above analysis, the selected genes were validated, more stringently filtered and applied to evaluate highly significant molecules. Interestingly, anterior gradient 2 (AGR2) was identified as a major gene highly correlated with survival rate and sorafenib resistance in liver cancer, but its associated mechanism and physiological significance have not been well elucidated. Therefore, AGR2 was selected for further study to investigate its molecular mechanism associated with sorafenib resistance and its physiological role in HCC.

AGR2 is a member of the protein disulfide isomerase (PDI) family of endoplasmic reticulum (ER) proteins that catalyze thiol-disulfide interchange and protein folding reactions [11, 12]. AGR2 was first identified in the estrogen receptor-expressing MCF-7 breast cancer cell line and was found to be regulated by estrogen both in vitro and in vivo [13–15]. AGR2 has been detected in different cancer types and is highly expressed in liver, breast, pancreas and bladder cancer tissues compared with healthy tissues [16]. Although AGR2 is an ER-resident protein, it has also been observed in the nucleus [17], cytoplasm [18], mitochondria [19], cell surface [20], extracellular matrix [21], urine [22] and blood [21]. Recently, Delom et al. showed that AGR2 localized in the extracellular matrix makes cancer cells more aggressive [23]. Moreover, AGR2 dysregulation has been implicated in certain disease processes, such as cancer progression and drug resistance [24]. Arumugam et al. reported that recombinant AGR2 can enhance pancreatic ductal adenocarcinoma cell migration, invasion and proliferation through C4.4a cell surface receptor-mediated signaling [25]. siRNA-mediated AGR2 knockdown induces cell death, inhibits cell growth and arrests cell cycle progression in breast cancer cells [26]. Similarly, AGR2 promotes cell growth and migration through the Akt signaling pathway in non-small cell lung cancer; moreover, the phospho-Akt level is reduced after depletion of AGR2 [27]. EGFR signaling is triggered by high AGR2 expression, which is defined as an early initiating factor and serves as a potential target for the curative treatment of neoplastic and chronic pancreatic disease [28]. Based on the above evidence, we suggest that AGR2 expression might be a marker to predict the presence of drug resistance in individual patients.

Previously, AGR2 was defined as a dominant factor in ER homeostasis [29]. Under ER stress, a series of adaptive mechanisms, such as unfolded protein response (UPR) signaling, are activated to cope with increased protein folding in the ER [30]. ER stress and UPR signaling activation result in the development and progression of several human diseases, including cancer [30]. Moreover, AGR2 has been demonstrated to be modulated by the UPR, likely through the PERK, ATF6, and IRE1α arms of the UPR, which can regulate the ER-associated degradation machinery (ERAD), resulting in induction of the cell’s ability to resolve ER stress [29]. Therefore, we suggest that AGR2 plays a critical role in the regulation of UPR signaling and ER stress.

In the present study, we found that AGR2 was highly correlated with overall and recurrence-free survival rates and with several clinical parameters in liver cancer. AGR2 was more highly expressed in sorafenib-resistant cells than in sorafenib-sensitive cells, and AGR2 was downregulated by sorafenib in both cell lines. Sorafenib-resistant cells were more tolerant to sorafenib and exhibited a lower apoptosis rate than sorafenib-sensitive cells. Sorafenib-induced cell death in HCC was found to be reversed and induced with recombinant AGR2 and AGR2-silencing constructs, respectively. The diverse regulatory mechanisms involved in sorafenib-induced cell death in both sorafenib-sensitive and sorafenib-resistant cells may be mediated by the modulation of ER stress and X-box binding protein (XBP) 1 status. This is the first report to uncover the molecular mechanism involved in sorafenib resistance, and further elucidation of the predictive role and molecular and cellular mechanisms of AGR2 related to sorafenib resistance may provide additional opportunities to establish complementary therapies for HCC.

## Materials and methods

### Database analysis

The two datasets, sorafenib-resistant dataset for Huh7 cells (GSE94550, [9]) and the Roessler Liver microarray dataset (GSE14520, [10]) were retrieved, and utilized > 2-fold change as the criterion for selection of sorafenib-modulated molecules in sorafenib-resistant Huh7 cells compared to parental cells (GSE94550) and > 1.2-fold as the criterion for choosing oncogenes related to survival in the bottom 25% vs. the top 25% of HCC patients from the Roessler Liver microarray dataset (GSE14520) to intersect the potential candidates.

### Cell culture and treatments

The HepG2, Huh7, J7 and Hep3B human hepatoma cell lines were routinely cultured in Dulbecco’s modified Eagle’s medium (DMEM; Invitrogen, Grand Island, NY) supplemented with 10% fetal bovine serum (HyClone, Road Logan, UT), 100 U/ml penicillin and 100 mg/ml streptomycin at 37 °C with 5% CO2. The hepatoma cells were stimulated with 0–10 μM sorafenib (Sigma–Aldrich, Burlington, MA).

### Quantitative RT-PCR

The cDNA template was prepared and Quantitative PCR reaction mixture contains 500 nM forward and reverse primers, and 1 × SYBR Green reaction mix (Applied Biosystems, Waltham, MA). SYBR Green fluorescence was determined by the ABI PRISM 7500 detection system (Applied Biosystems). AGR2 Forware-5′ GAGCCAAAAAGGACACAAAGGA 3′, Reverse-5′ TGAGTTGGTCACCCCAACCT 3′; 18SrRNA Forware-5'GCAGCTCACCTACCTGGAGAAATA3′, Reverse 5'TGCGTGTGTGGGTCTTTGAA3′.

### Immunohistochemistry

The use of archived formalin-fixed, paraffin-embedded tissue blocks was approved by the Institutional Review Board of National Cheng Kung University Hospital. Tissue slides from HCC patients were evaluated via immunohistochemistry and hematoxylin/eosin staining using a polyclonal antibody against AGR2 (GeneTex, Hsinchu, Taiwan) according to the avidin–biotin complex method, as described previously [31]. Immunoreactivity for AGR2 was visualized using DAB/nickel substrate (Vector Laboratories, Burlingame, CA).

### MTT assay

Cell viability was analyzed using 3-(4,5-dimethylthiazol-2-yl)-2,5-diphenyltetrazolium bromide (MTT) assay(Sigma–Aldrich, Burlington, MA). Cells (5 × 10^3^) were seeded on 96-well plates overnight. After treatment, 20 μl MTT reagent was added to each well for 3 h, and the absorbance at 570 nm was determined with a SpectraMax microplate reader.

### Apoptosis assay

At the end of treatment, the cells were washed and resuspended in binding buffer (Annexin V PE Apoptosis Detection kit; BD Biosciences, East Rutherford, NJ). After incubation with annexin V-PE and propidium iodine (PI), binding buffer was added, and the cells were analyzed via fluorescence-activated cell sorting (FACScan, BD Biosciences). Data analysis was performed using Cell Quest software.

### RT–PCR

The cDNA template was prepared and amplified via PCR for 30 cycles at 95 °C for 15 s, 54 °C for 15 s, and 72 °C for 30 s (AGR2) or for 33 cycles at 95 °C for 15 s, 57 °C for 15 s, and 72 °C for 30 s (XBP1). 18S rRNA was used as an internal control. PCR products were examined via 2% agarose gel (Amresco, Solon, OH, USA) electrophoresis. The following primers were used: AGR2 Forward-5′ GAGCCAAAAAGGACACAAAGGA 3′, Reverse-5′ TGAGTTGGTCACCCCAACCT 3′; XBP1 Forward-5′ TTACGAGAGAAAACTCATGGCC 3′, Reverse-5′ GGGTCCAAGTTGTCCAGAATGC 3′. 18S rRNA Forward-5′ GCAGCTCACCTACCTGGAGAAATA 3′, Reverse-5′ TGCGTGTGTGGGTCTTTGAA 3′.

### Western blotting

Total protein was fractionated via 8–12% SDS–PAGE, transferred to an immobilon polyvinylidene difluoride membrane (Amersham Biosciences, Amersham, UK), and hybridized with specific primary antibodies against AGR2 (GeneTex, Hsinchu, Taiwan), ATF6 (GeneTex), p-IRE1α (GeneTex), IRE1α (Cell Signaling Technology, Danvers, MA), p-PERK (GeneTex), PERK (Cell Signaling Technology) and β-actin (GeneTex) overnight at 4 °C. Subsequently, the membrane was probed with the appropriate HRP-conjugated secondary antibody for 1 h at room temperature. Finally, immune complexes were visualized via the chemiluminescence method using an ECL detection kit (Merck, Darmstadt, Germany).

### Establishment of AGR2-silenced cells

Small hairpin (sh) RNA and small interfering (si) RNA targeting AGR2 were purchased from Academia Sinica and Thermo Fisher Scientific. HepG2 and Huh7 cells were transfected with siRNA and/or shRNA targeting AGR2 using Lipofectamine 2000 reagent (Invitrogen). After transfection, the expression of AGR2 was determined using RT–PCR and Western blotting.

### Establishment of sorafenib-resistant HCC cells

HepG2 and Huh7 hepatoma cells were cultured in medium containing increasing concentrations of sorafenib in the range of 0.5–7 μM over a period of 6 months. After successful establishment, sorafenib-resistant HepG2 (SR-HepG2) and Huh7 (SR-Huh7) cells were maintained in medium containing 7 μM sorafenib. The sorafenib-resistant cells were routinely cultured in DMEM medium containing sorafenib. For various experiments, the parental and sorafenib-resistant cells were seeded in the regular DMEM medium without sorafenib to attenuate the sorafenib effect in the routinely cultured sorafenib-resistant cells. After seeding 24 h, the parental and sorafenib-resistant cells were then treated with the indicated concentrations (5–10 μM) and time points (24–48 h) of sorafenib. At the end of treatment, the cell lysate and conditioned medium were collected.

### Preparation of conditioned medium (CM)

CM was collected and centrifuged at 2000 × *g* for 5 min to eliminate intact cells, concentrated in spin columns with a 3-kDa molecular weight cutoff (Amicon Ultra, Millipore), and stored at − 80 °C for subsequent experiments.

### Clinical HCC specimens

HCC tissues were obtained from the National Health Research Institute Biobank and the human biobank of National Cheng Kung University Hospital. All clinical specimen experiments were performed in accordance with the guidelines of the Institutional Review Board of National Cheng Kung University Hospital (IRB No: A-ER-109-533). Informed consent was waived for the use of the specimens from the National Health Research Institute Biobank and human biobank of National Cheng Kung University Hospital.

### Statistical analysis

Correlations between the AGR2 level (Roessler liver array, 39-△Ct) and clinicopathological indicators were assessed using a Wilcoxon rank sum test. Data are presented as the mean ± SD. Recurrence-free survival (RFS) and overall survival (OS) were calculated using the Kaplan–Meier method, and a log-rank test was used to assess differences between groups. A Cox proportional hazards regression model was used to measure the independence of different factors. Cox regression was performed via forward stepwise analysis, and only the prognostic variables that were significant in the univariate analysis were included in the model. All values are reported as the mean ± SD. Two-way ANOVA, Student’s t test, a chi-square test, or Fisher’s exact test was applied to evaluate experimental differences among groups. p values less than 0.05 were considered to indicate statistical significance.

## Results

### AGR2 is clinically relevant in HCC

**Fig. 1:**
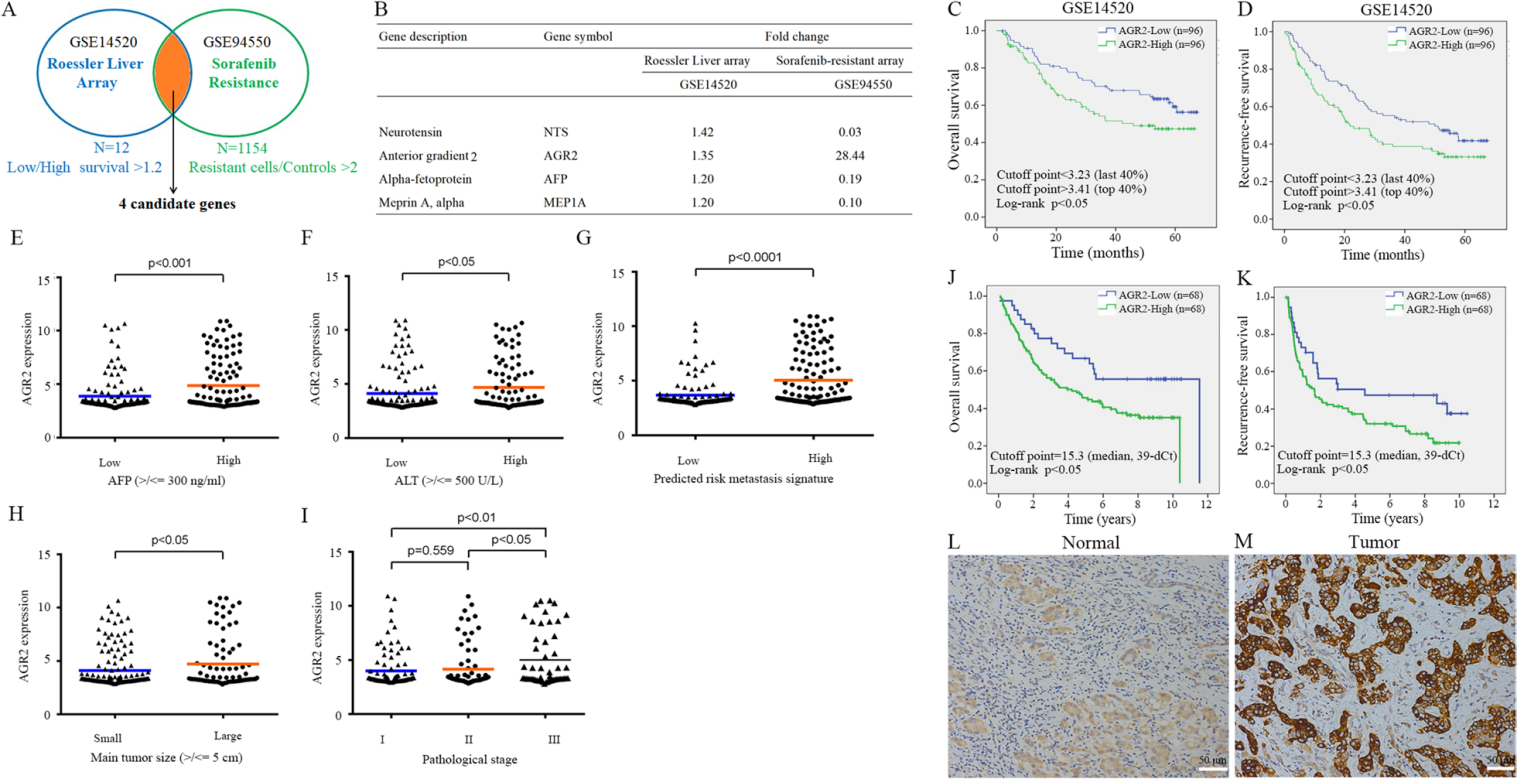
Selection of potential candidate genes and analysis of clinical parameter correlations and survival. **A** Schematic diagram of the analytic protocol for the selection of sorafenib-mediated candidate genes. The 4 selected candidate genes were consistently observed in these two datasets. **B** The gene descriptions and fold changes in the GSE14520 and GSE94550 datasets are shown. **C**–**D**, **J**–**K** KM survival curves (OS and RFS) of 2 groups of patients with HCC grouped by AGR2 expression level [cutoff established on the basis of Roessler liver microarray scores] (C-D, GSE14520) and the cycle threshold value (Ct) obtained by quantitative PCR in our collected HCC specimens (**J**–**K**, median, 39-dCt). Patients with high AGR2 levels have worse overall survival and recurrence-free survival. **E**–**I** The AGR2 levels with several parameter correlations are shown. **L**, **M** Immunohistochemistry staining showing AGR2 expression in HCC specimens (200X). AGR2 is highly expressed in tumor tissues compared with normal liver tissue. d: delta

We retrieved two datasets, the sorafenib-resistant dataset for Huh7 cells (GSE94550, [9]) and the Roessler Liver microarray dataset (GSE14520, [10]), and utilized > 2-fold change as the criterion for selection of sorafenib-modulated molecules in sorafenib-resistant Huh7 cells compared to parental cells (GSE94550) and > 1.2-fold as the criterion for choosing oncogenes related to survival in the bottom 25% vs. the top 25% of HCC patients from the Roessler Liver microarray dataset (GSE14520) to intersect the potential candidates for further study. According to the above analysis, the selected genes were validated and filtered more stringently and applied to evaluate highly significant molecules. After retrieving these datasets, 545 upregulated genes (sorafenib resistance vs. control >2-fold) and 609 downregulated genes (sorafenib resistance vs. control < 2-fold) were identified in the sorafenib-resistant hepatoma Huh7 cell dataset (GSE94550). In the Roessler Liver microarray dataset (GSE14520), we used > 1.2-fold as the criterion for choosing oncogenes related to survival in the bottom 25% vs. the top 25% of HCC patients, and only 12 dysregulated genes were identified (Fig. 1A). Finally, we identified 4 potential candidates in the two intersecting microarray datasets, namely, neurotensin (NTS), AGR2, alpha-fetoprotein (AFP) and meprin A, alpha (MEP1A) (Fig. 1A, B). Previously, the AGR2 protein was demonstrated to be a part of the PDI family of ER proteins that mediate the formation of disulfide bonds and catalyze protein folding [32]. Moreover, AGR2 is highly expressed in numerous cancer types, including liver cancer [16]. Through a clinical study, Hrstka et al. showed that AGR2 expression can be used as a marker to predict poor prognosis in breast cancer [15]. Hence, we suggest that AGR2 may be a potential candidate target in sorafenib-treated HCC. Interestingly, AGR2 is the major gene highly correlated with the survival rate and sorafenib resistance in liver cancer; however, the relationship between AGR2 and sorafenib treatment in HCC has not been demonstrated. Therefore, AGR2 was selected for further study to investigate its molecular mechanism associated with sorafenib resistance and its physiological role in HCC.

The clinical significance of AGR2 expression was analyzed in the Roessler Liver database (GSE14520) and in our collected cohort (Fig. 1C–K). Patients with lower AGR2 expression (last 40%) had a better OS rate (log-rank *P* < 0.05; AGR2 high (top 40%): standard error, 2.738; 95% CI 36.196–46.927; AGR2 low (last 40%): standard error, 2.432; 95% CI 45.322–54.857) and RFS rate (log-rank *P* < 0.05; AGR2 high (top 40%): standard error, 2.766; 95% CI 27.398–38.243; AGR2 low (last 40%): standard error, 2.645; 95% CI 36.365–46.734) (Fig. 1C, D). The Roessler Liver microarray dataset (GSE14520) provides detailed clinical information; therefore, the correlation between AGR2 and various clinical parameters was statistically analyzed to define the role of sorafenib-regulated AGR2 in HCC progression (Tables 1–3). High AGR2 expression was significantly correlated with high AFP and ALT levels, a high predicted risk metastasis signature score (Fig. 1E, F, G), a large primary tumor size and more advanced pathological stages of HCC (Fig. 1H, I). A high AGR2 level in HCC was correlated with a high serum AFP level (P = 0.002) (Table 1). Univariate analysis showed that male sex (P = 0.009), tumor size (P = 0.045), CLIP score (P = 0.002), BCLC stage (P < 0.001), AJCC stage (P < 0.001), and high AGR2 level (P = 0.003) were significant predictors of worse RFS (Table 2). Multivariate analysis showed that male sex (P = 0.023, HR = 2.124, CI = 1.112–4.058), BCLC stage (P = 0.021, HR = 1.653, CI = 1.077–2.535), AJCC stage (P = 0.019, HR = 1.607, CI = 1.083–2.386) and a high AGR2 level (P = 0.010, HR = 1.572, CI = 1.112–2.224) were independently associated with RFS (Table 2). For OS, univariate analysis showed that cirrhosis (P = 0.023), AFP level (P = 0.011), tumor size (P = 0.001), CLIP score (P < 0.001), BCLC stage (P < 0.001), AJCC stage (P < 0.001), and a high AGR2 level (P = 0.002) were significant predictors of worse OS (Table 3). Multivariate analysis showed that cirrhosis (P = 0.034, HR = 4.563, CI = 1.122–18.555), BCLC stage (P < 0.001, HR = 2.928, CI = 1.908–4.494), and a high AGR2 level (P = 0.008, HR = 1.735, CI = 1.154–2.609) were independently associated with OS (Table 3).

**Table 1:** Association of AGR2 level (Roessler liver array) with clinicopathologic indicators of hepatocellular carcinoma

Factors	Group	AGR2 (mean ± SE)	P
Age	< 60 years	4.3208 ± 0.1474	0.900
	≥ 60 years	4.3935 ± 0.3107	
Sex	Male	4.3334 ± 0.1422	0.926
	Female	4.3525 ± 0.3850	
Cirrhosis	Absent	3.6259 ± 0.3076	1.162
	Present	4.3963 ± 0.1415	
Serum AFP	< 300	3.8669 ± 0.1446	0.002*
(ng/ml)	≥ 300	4.8537 ± 0.2261	
Tumor size	< 5 cm	4.1165 ± 0.1462	0.055
	≥ 5 cm	4.7315 ± 0.2591	
CLIP	0–1	4.2594 ± 0.1429	0.420
	≥ 2	4.6445 ± 0.3413	
BCLC	0–1	4.1908 ± 1.9342	0.094
	≥ 2	4.8530 ± 2.4513	
AJCC stage	I	4.1535 ± 1.8291	0.381
	≥ II	4.4955 ± 2.2573	

^*^P < 0.05. CLIP, Cancer of the Liver Italian Program score; BCLC, Barcelona Clinic Liver Cancer staging; AJCC, American Joint Committee on Cancer 2017; AFP, alpha-fetoprotein

**Table 2:** Prognostic significance of clinicopathologic indicators and AGR2 for recurrence-free survival in the Roessler liver array

Factor	RFS univariate				RFS multivariate		
	Group	HR	95% CI	P	HR	95% CI	P
Age	< 60/ ≥ 60 years	0.952	0.628–1.443	0.817			
Sex	Female/Male	2.359	1.238–4.493	0.009*	2.124	1.112–4.058	0.023*
Cirrhosis	−/ +	2.003	0.936–4.287	0.074			
Serum AFP	< 300/ ≥ 300 ng/ml	1.314	0.937–1.842	0.113			
Tumor size	< 5/ ≥ 5 cm	1.424	1.008–2.012	0.045*			NS
CLIP	0–1/ ≥ 2	1.872	1.267–2.766	0.002*			NS
BCLC	0–1/ ≥ 2	2.432	1.670–3.543	< 0.001*	1.653	1.077–2.535	0.021*
AJCC stage	I/ ≥ II	2.063	1.405–3.029	< 0.001*	1.607	1.083–2.386	0.019*
AGR2	Low/High	1.691	1.202–2.378	0.003*	1.572	1.112–2.224	0.010*

^*^P < 0.05. RFS, recurrence-free survival; CLIP, Cancer of the Liver Italian Program score; BCLC, Barcelona Clinic Liver Cancer staging; AJCC, American Joint Committee on Cancer 2017; AFP, alpha-fetoprotein

**Table 3:** Prognostic significance of clinicopathologic indicators and AGR2 for overall survival in the Roessler liver array

Factor	RFS univariate				RFS multivariate		
	Group	HR	95% CI	P	HR	95% CI	P
Age	< 60/ ≥ 60 years	0.990	0.972–1.008	0.990			
Sex	Female/Male	1.858	0.901–3.833	0.094			
Cirrhosis	−/ +	5.093	1.255–20.671	0.023*	4.563	1.122–18.555	0.034*
Serum AFP	< 300/ ≥ 300 ng/ml	1.686	1.126–2.527	0.011*			
Tumor size	< 5/ ≥ 5 cm	1.960	1.309–2.933	0.001*			
CLIP	0–1/ ≥ 2	2.811	1.832–4.313	< 0.001*			
BCLC	0–1/ ≥ 2	3.176	2.081–4.846	< 0.001*	2.928	1.908–4.494	< 0.001*
AJCC stage	I/ ≥ II	2.278	1.483–3.500	< 0.001*			
AGR2	Low/high	1.919	1.282–2.873	0.002*	1.735	1.154–2.609	0.008*

^*^P < 0.05. OS, overall survival; CLIP, Cancer of the Liver Italian Program score; BCLC, Barcelona Clinic Liver Cancer staging; AJCC, American Joint Committee on Cancer 2017; AFP, alpha-fetoprotein

Moreover, our HCC specimen cohort was also analyzed. We utilized qRT–PCR to examine the levels of AGR2, and a median level of 15.3 (39-△Ct) was defined as the cutoff to divide the HCC specimens into high and low AGR2 expression groups. Similar results were observed; high AGR2 levels were related to significantly worse OS and RFS rates (Fig. 1J, K). Furthermore, the correlation between AGR2 expression and various clinical parameters in our collected cohort was analyzed (Tables 4–6). A higher AGR2 level in HCC was observed in female patients (P = 0.007) (Table 4). A high AGR2 level (39-△Ct ≥ 10.8) in HCC was significantly associated with worse OS (P = 0.016) (Fig. 1J) and RFS (P = 0.045) (Fig. 1K). Univariate analysis showed that cirrhosis (P = 0.022), AFP level (P = 0.033), vascular invasion (P = 0.003), AJCC stage (P = 0.003), and a high AGR2 level (P = 0.047) were significant predictors of worse RFS (Table 5). Multivariate analysis showed that cirrhosis (P = 0.023, HR = 1.600, CI = 1.067–2.401), vascular invasion (P = 0.005, HR = 1.825, CI = 1.198–2.780), and a high AGR2 level (P = 0.043, HR = 1.662, CI = 1.017–2.716) were independently associated with RFS (Table 5). For OS, univariate analysis showed that cirrhosis (P = 0.020), AFP level (P = 0.012), vascular invasion (P < 0.001), AJCC stage (P = 0.007), and a high AGR2 level (P = 0.018) were significant predictors of worse OS (Table 6). Multivariate analysis showed that cirrhosis (P = 0.017, HR = 1.655, CI = 1.093–2.505), vascular invasion (P = 0.001, HR = 2.200, CI = 1.392–3.476), and a high AGR2 level (P = 0.015, HR = 1.945, CI = 1.138–3.324) were independently associated with OS (Table 6).

**Table 4:** Association of AGR2 expression with clinicopathologic indicators of hepatocellular carcinoma

Factors	Group	AGR2 (mean ± SE)	P
Age	< 60 years	15.8713 ± 0.7077	0.659
	≥ 60 years	16.2616 ± 0.4603	
Sex	Male	15.4998 ± 0.4755	0.007*
	Female	17.6959 ± 0.5801	
Hepatitis viral	Absent	15.9186 ± 0.6085	0.812
infection	Present	16.2114 ± 0.4827	
Cirrhosis	Absent	16.1498 ± 0.4687	0.989
	Present	16.0949 ± 0.6798	
Serum AFP	< 200	15.8232 ± 0.5254	0.403
(ng/ml)	≥ 200	16.5543 ± 0.5651	
Tumor	W	14.4390 ± 1.5358	0.240
differentiation	M-P	16.2353 ± 0.3985	
Tumor size	< 5 cm	15.7190 ± 0.5735	0.482
	≥ 5 cm	16.3917 ± 0.5167	
Vascular	Absent	15.5317 ± 0.6740	0.198
invasion	Present	16.5114 ± 0.4624	
AJCC stage	I	15.3584 ± 0.8662	0.241
	≥ II	16.3968 ± 0.4242	

^*^P < 0.05. Tumor differentiation by WHO; AJCC, American Joint Committee on Cancer 2017; AFP, alpha-fetoprotein

**Table 5:** Prognostic significance of clinicopathologic indicators and AGR2 for recurrence-free survival in the clinical cohort

Factor	RFS univariate				RFS multivariate		
	Group	HR	95% CI	P	HR	95% CI	P
Age	< 60/ ≥ 60 years	1.079	(0.713–1.632)	0.720			
Sex	Male/female	1.059	(0.672–1.670)	0.804			
Viral infection				0.248			
	No/B	1.667	(0.977–2.844)	0.061			
	No / C	1.274	(0.739–2.915)	0.384			
	No / B + C	1.647	(0.835–3.262)	0.152			
Cirrhosis	-/ +	1.602	(1.069–2.401)	0.022*	1.600	(1.067–2.401)	0.023*
Serum AFP	< 200/ ≥ 200 ng/ml	1.548	(1.037–2.312)	0.033*			NS
Differentiation	W/M-P	3.053	(0.964–9.670)	0.058			
Tumor size	< 5/ ≥ 5 cm	1.161	(0.764–1.765)	0.484			
Vascular invasion	−/ +	1.899	(1.248–2.890)	0.003*	1.825	(1.198–2.780)	0.005*
AJCC stage	I/ ≥ II	2.100	(1.282–3.440)	0.003*			NS
AGR2	Low/high	1.638	(1.006–2.670)	0.047*	1.662	(1.017–2.716)	0.043*

^*^P < 0.05. DFS, disease-free survival; Tumor differentiation according to WHO system; AFP, alpha-fetoprotein; AJCC, American Joint Committee on Cancer 2017

**Table 6:** Prognostic significance of clinicopathologic indicators and AGR2 for overall survival in the clinical cohort

Factor	OS univariate				OS multivariate		
	Group	HR	95% CI	P	HR	95% CI	P
Age	< 60/ ≥ 60 years	0.975	(0.638–1.490)	0.907			
Sex	Male/female	1.407	(0.906–2.186)	0.936			
Viral infection				0.281			
	No/B	1.546	(0.890–2.685)	0.122			
	No / C	1.014	(0.573–1.794)	0.962			
	No / B + C	1.433	(0.704–2.916)	0.322			
Cirrhosis	−/ +	1.632	(1.080–2.466)	0.020*	1.655	(1.093–2.505)	0.017*
Serum AFP	< 200/ ≥ 200 ng/ml	1.692	(1.122–2.551)	0.012*			NS
Differentiation	W/M-P	1.330	(0.851–2.079)	0.210			
Tumor size	< 5/ ≥ 5 cm	1.101	(0.720–1.683)	0.658			
Vascular invasion	−/ +	2.302	(1.458–3.635)	< 0.001*	2.200	(1.392–3.476)	0.001*
AJCC stage	I/ ≥ II	2.076	(1.224–3.521)	0.007*			NS
AGR2	Low/high	1.894	(1.114–3.221)	0.018*	1.945	(1.138–3.324)	0.015*

^*^P < 0.05. OS, overall survival; Tumor differentiation according to WHO system; AFP, alpha-fetoprotein; AJCC, American Joint Committee on Cancer 2017

Moreover, immunohistochemical staining was utilized to examine the expression of AGR2 in 24 clinical HCC tissues, and the results indicated that AGR2 was highly expressed in tumor tissues compared to normal tissues (Fig. 1L, M). Overall, based on the evidence, we found that patients with lower AGR2 expression have a better OS rate; hence, we suggest that AGR2 might play an oncogenic role in HCC progression and thus might be a useful prognostic marker of HCC progression.

### Sorafenib decreases cell viability and increases cell apoptosis

**Fig. 2:**
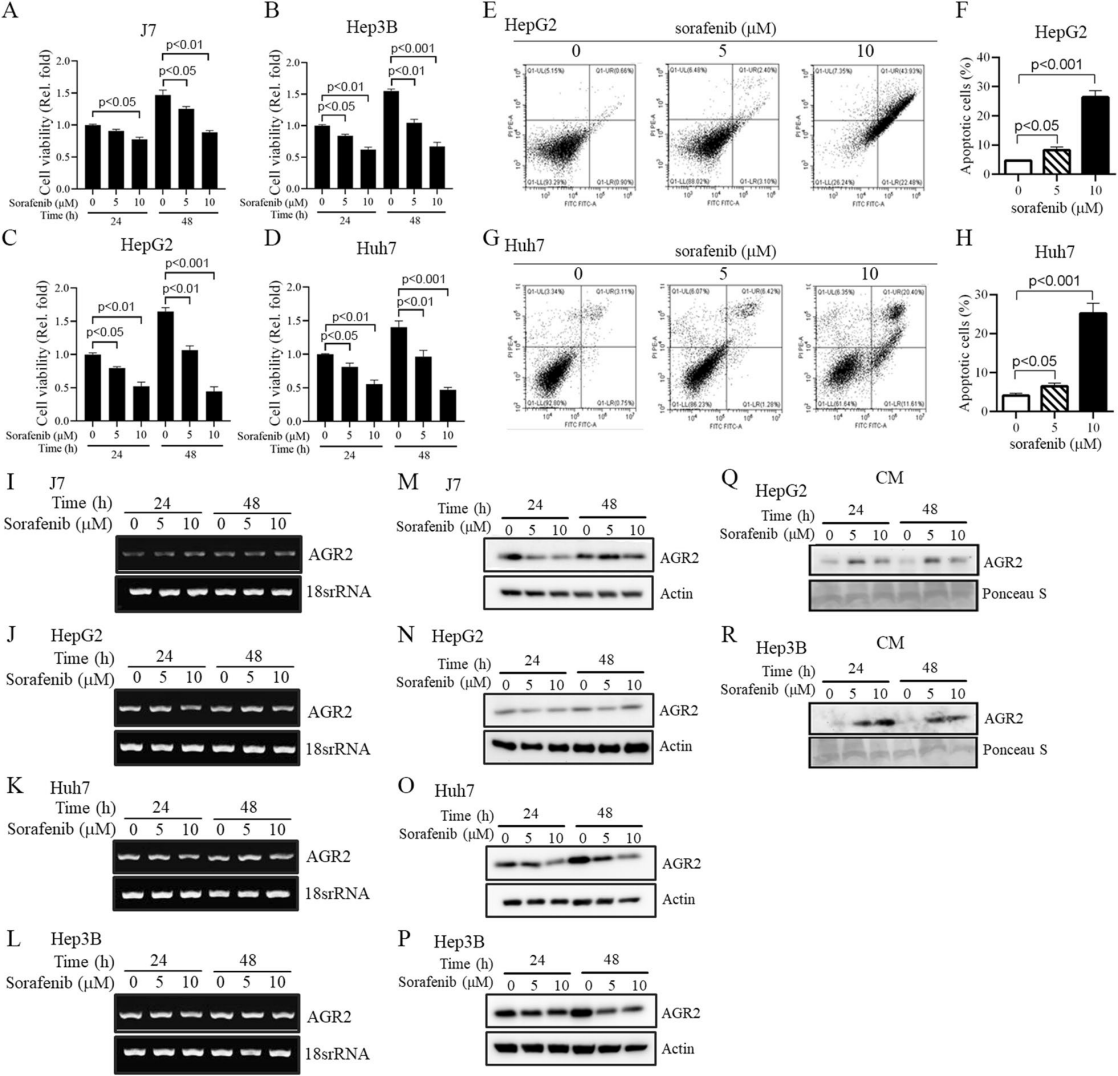
Sorafenib decreases cell viability, increases cell apoptosis and induces AGR2 secretion in HCC. **A**–**D** The viability of J7, Hep3B, HepG2, and Huh7 HCC cells treated with 5 and 10 μM sorafenib for 24–48 h was examined using MTT assay. **E**–**H** Cell apoptosis was determined in HepG2 and Huh7 cells after stimulation with 5 and 10 μM sorafenib. The quantification of apoptotic cells is shown in **F**, **H**. Sorafenib decreases cell viability and increases cell apoptosis in HCC cells. **I**–**R** AGR2 RNA **I**–**L** and protein **M**–**R** levels, both intracellular **M**–**P** and extracellular **Q**, **R**, were examined via RT–PCR and Western blotting after 5 and 10 μM sorafenib treatment for 24–48 h. Sorafenib induces AGR2 secretion from the cytosol into conditioned medium rather than exerting transcriptional or translational regulation (lane 1, 4:untreatment; lane 2, 3, 5, 6:sorafenib treatment). Ponceau S was used as an internal control

First, to determine the effect of sorafenib on cell viability, HCC cell lines were treated with various doses of sorafenib (5–10 μM, 24–48 h). Cell viability was significantly decreased with sorafenib treatment in a dose-dependent manner in J7, Hep3B, HepG2 and Huh7 cells according to MTT assay results (Fig. 2A–D). Moreover, flow cytometry was utilized to determine whether sorafenib influences HCC cell apoptosis. HepG2 and Huh7 cells were stimulated with 5 and 10 μM sorafenib for 24 h. Cell apoptosis was slightly induced with 5 μM sorafenib; however, the increase in the apoptosis rate compared with that in the control reached approximately 25% after 10 μM sorafenib stimulation in both HepG2 and Huh7 cells (Fig. 2E–H). Based on these results, we found that sorafenib can modulate HCC cell viability and apoptosis ability; subsequently, we evaluated whether AGR2 is involved in sorafenib-regulated phenotypes.

### Sorafenib induces AGR2 secretion instead of transcriptional regulation

After we retrieved the GSE94550 dataset, AGR2 was found to be induced in sorafenib-resistant Huh7 cells compared to parental cells (Fig. 1A). To further examine whether sorafenib can regulate AGR2 expression in HCC, RT–PCR and Western blotting were applied in parental HCC cell lines treated with sorafenib. First, we found that the RNA level of AGR2 was not altered by sorafenib in J7, HepG2, Huh7 and Hep3B cells (Fig. 2I-L; Additional file 1: Figure S1A–D). However, the protein level of AGR2 in the lysates of these cells was unexpectedly decreased in a dose-dependent manner after sorafenib stimulation, as shown by Western blotting (lane 3 vs 1, lane 6 vs 4; Fig. 2M–P; Additional file 1: Figure S1E–H). Based on these contradictory results, sorafenib regulated the RNA and protein levels of AGR2 in parental HCC cells. Several reports have shown that AGR2 is an ER-resident protein that is also localized in the extracellular matrix, blood and urine [21, 22]. Therefore, we collected CM after 5 and 10 μM sorafenib treatments for 24–48 h. As expected, AGR2 was detected in CM from HepG2 and Hep3B cells treated with sorafenib (lane 2, 3 vs 1, lane 5, 6 vs 4; Fig. 2Q, R; Additional file 1: Figure S1I-J). Based on this evidence, we suggest that sorafenib regulates AGR2 through posttranslational modification, not transcriptional regulation, in parental HCC cells.

### AGR2 plays a role in cell viability and apoptosis

**Fig. 3:**
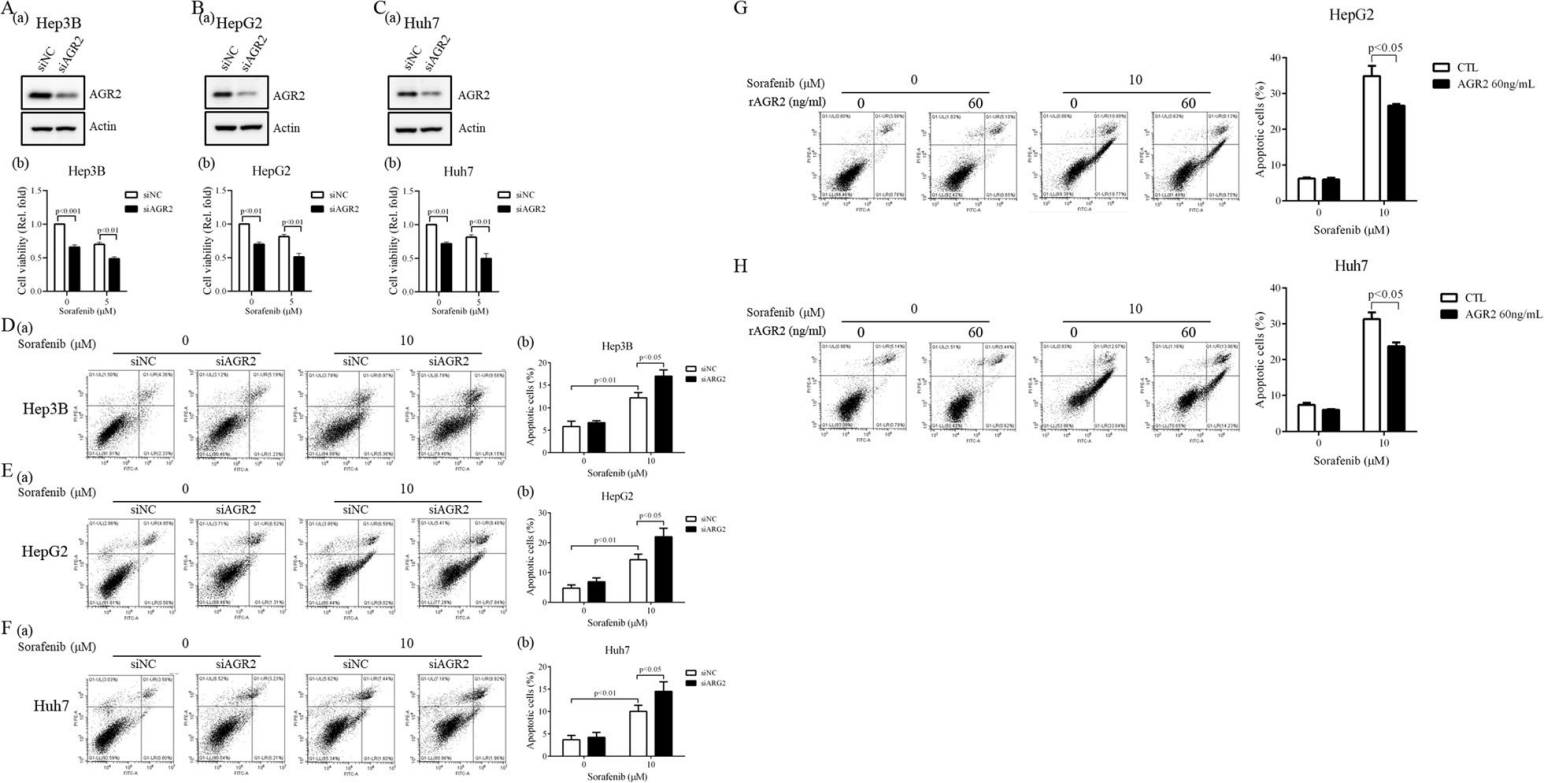
AGR2 is implicated in sorafenib-regulated cell viability and apoptosis. **A**–**C** The viability of Hep3B, HepG2, and Huh7 cells with silenced AGR2 was examined after 24 h in the presence or absence of sorafenib (5 μM) using MTT assay. The AGR2 levels (**A**(a), **B**(a), **C**(a)) and cell viability (**A**(b), **B**(b), **C**(b)) results are presented. AGR2 supports HCC cell viability. **D**–**H** HCC cell apoptosis was assessed after AGR2 silencing **D**–**F** or stimulation with 60 ng/ml recombinant AGR2 (rAGR2) **G**, **H** in the presence of 10 μM sorafenib. The quantification of apoptotic cells is shown (**D**(b), **E**(b), **F**(b)). AGR2 inhibition, using either siRNA transfection or recombinant protein treatment, decreases cell apoptosis. siNC: negative control siRNA, siRNA vector only; siAGR2: AGR2 siRNA

To analyze the roles of AGR2 in HCC progression, we established AGR2-silenced Hep3B, HepG2 and Huh7 cells (lane 2 vs 1; Fig. 3A(a), B(a), C(a); Additional file 2: Figure S2), and cell viability was determined using MTT assay. Cell viability was significantly decreased after AGR2 silencing, and the effect was more obvious when AGR2 silencing was combined with sorafenib treatment (Fig. 3A(b), B(b), C(b)). These findings suggest that AGR2 plays a role in promoting cancer progression. Additionally, we investigated whether AGR2 affects cell apoptosis. Flow cytometry analysis was utilized to demonstrate that sorafenib can induce significant cell apoptosis (approximately 10%) in Hep3B, HepG2 and Huh7 cells compared with control cells, which was more conspicuous after AGR2 silencing in the presence of sorafenib compared to the control (siNC) (Fig. 3D, E, F). Quantitative results are shown in panels 3D (b), E (b), and F (b). Apoptotic cells appeared among the siNC cells under untreated conditions, and we speculate that apoptosis might have been induced in these cells by the transfection process or the apoptosis assay procedures. However, the phenomenon of sorafenib-treated or AGR2-silenced cell apoptosis was not influenced by the basal apoptosis signal in untreated cells. The sorafenib-treated and AGR2-silenced cell apoptosis rates were normalized to the value in the untreated basal cells. However, we found that AGR2 can be secreted into CM and detected by Western blotting (CM, Fig. 2Q, R). Previously, Fessart et al. reported that extracellular AGR2 can be defined as an extracellular matrix pro-oncogenic regulator that makes cancer cells more aggressive [21]. Hence, we evaluated whether the addition of recombinant AGR2 protein regulates cell viability and apoptosis. Flow cytometry analysis indicated that 10 μM sorafenib can induce up to 30–40% cell apoptosis at 24 h in both HepG2 and Huh7 cells; however, the phenomenon was reversed with 60 ng/ml recombinant AGR2 (rAGR2) (Fig. 3G, H). Based on this evidence, we suggest that AGR2 secreted into CM in response to sorafenib stimulation plays an oncogenic role in HCC cancer progression.

### Sorafenib induces ER stress in HCC

**Fig. 4:**
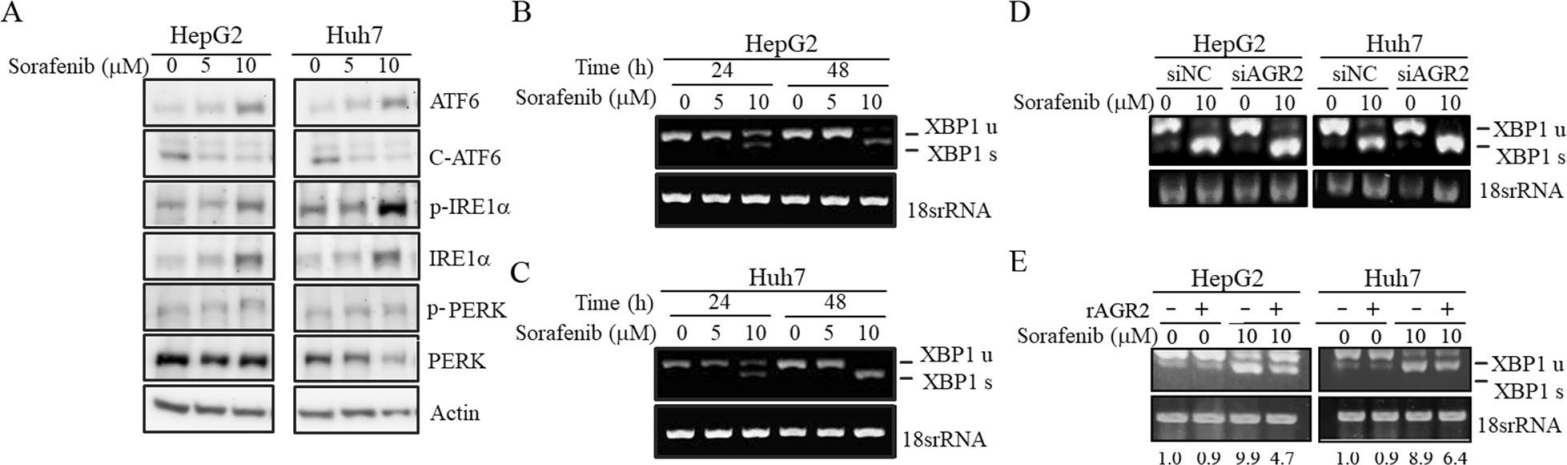
Sorafenib regulates ER stress-related molecules. **A**–**E** The protein **A** and RNA **B**, **C**, **D, E** levels of ER stress-related molecules were examined by Western blotting **A** and RT–PCR **B**, **C**, **D**, **E** after treatment of HepG2 **A**, **D**, **E** (left), **B** and Huh7 **A**, **D**, **E** (right), **C** cells with 5 μM and 10 μM sorafenib with or without AGR2 silencing **D** and 60 ng/ml recombinant AGR2 (rAGR2) stimulation **E**. Sorafenib induces the dysregulation of several ER stress-related molecules. AGR2 is involved in sorafenib-induced XBP-1 splicing. C: cleavage; u: unspliced; s: spliced; siNC: vector control siRNA; siAGR2: AGR2 siRNA. 0: untreatment; 5, 10: sorafenib treatment

We determined the functions and regulatory mechanisms through which sorafenib influences AGR2 activity. Based on a literature search, AGR2 has been demonstrated to be upregulated upon ER stress, and ER stress-related molecules, such as protein kinase R (PKR)-like endoplasmic reticulum kinase (PERK), inositol-requiring enzyme 1 (IRE1) and activating transcription factor 6 (ATF6), are dysregulated in many cancer types [16]. Therefore, the relationship between sorafenib and ER-related factors was determined. Among these molecules, using Western blotting, we found that phospho-IRE1α (p-IRE1α) was upregulated by 10 μM sorafenib treatment in HepG2 and Huh7 cells (lane 3 vs 1; Fig. 4A; Additional file 3: Figure S3). The Bip protein level was determined using Western blotting after sorafenib treatments at numerous concentrations in J7 and Huh7 cells. However, Bip expression was not altered by sorafenib (lane 2, 3 vs 1; Additional file 4: Figure S4). Moreover, X-box binding protein 1 (XBP1) has been reported as a unique transcription factor that modulates ERAD gene expression and promotes protein folding [33]. In the UPR, IRE1α is activated via oligomerization and autophosphorylation, followed by the activation of its endoribonuclease to cleave and splice XBP1. The activated IRE1α endoribonuclease can remove 26 nucleotides from the intron of XBP1, converting XBP1 from preform XBP1 (XBP1 u: unspliced) to activated XBP1 (XBP1 s: spliced) [34]. Therefore, RT–PCR was used to detect the status of the IRE-1 downstream factor XBP1. Through RT–PCR analysis, we found that XBP1 was spliced from inactive XBP1 u to active XBP1 s after stimulation with sorafenib for 24 and 48 h in HepG2 and Huh7 cells (lane 3 vs 1, lane 6 vs 4; Fig. 4B, C; Additional file 5: Figure S5A, B). The data showed two bands: the upper band is full-length (unspliced, u) XBP1 (XBP1 u), and the lower band is spliced (s) XBP1 (XBP1 s). Therefore, we found that spliced XBP1 was increased and unspliced XBP1 was decreased after sorafenib treatment for 24 and 48 h. Subsequently, we sought to verify whether AGR2 plays a role in the conversion from inactive XBP1 u to active XBP1 s. As expected, the sorafenib-induced XBP1 s levels were more robust in HepG2 and Huh7 cells with silenced AGR2 (siAGR2) compared with vector control (siNC) under sorafenib treatment (lane 4 vs 2; Fig. 4D; Additional file 5: figure S5C, D). In contrast, we further verified whether added recombinant AGR2 (rAGR2) could modulate the splicing of inactive XBP1 u to activate XBP1 s. Similarly, the levels of XBP1 s induced by 10 μM sorafenib were reduced after stimulation with 60 ng/ml rAGR2 in HepG2 and Huh7 cells (lane 4 vs 3; Fig. 4E; Additional file 5: figure S5E, F). Moreover, we analyzed AGR2 and XBP1 expression in our HCC clinical specimen cohort using RT‒PCR. We analyzed 9 normal liver tissues and 5 HCC tumor tissues. AGR2 was slightly more highly expressed in HCC tumor tissues than in normal tissues, and the spliced XBP1 (XBP1 s) level was slightly more highly expressed in HCC tissues. We suggest that the correlation between AGR2 and XBP1 needs to be further demonstrated in more clinical specimens in the future (Normal: lanes 1–9, Tumor: lanes 1–5; Additional file 6: figure S6). Collectively, these findings indicate that sorafenib induces HCC ER stress via the IRE1α-XBP1 cascade through AGR2 regulation.

### AGR2 plays diverse roles

**Fig. 5:**
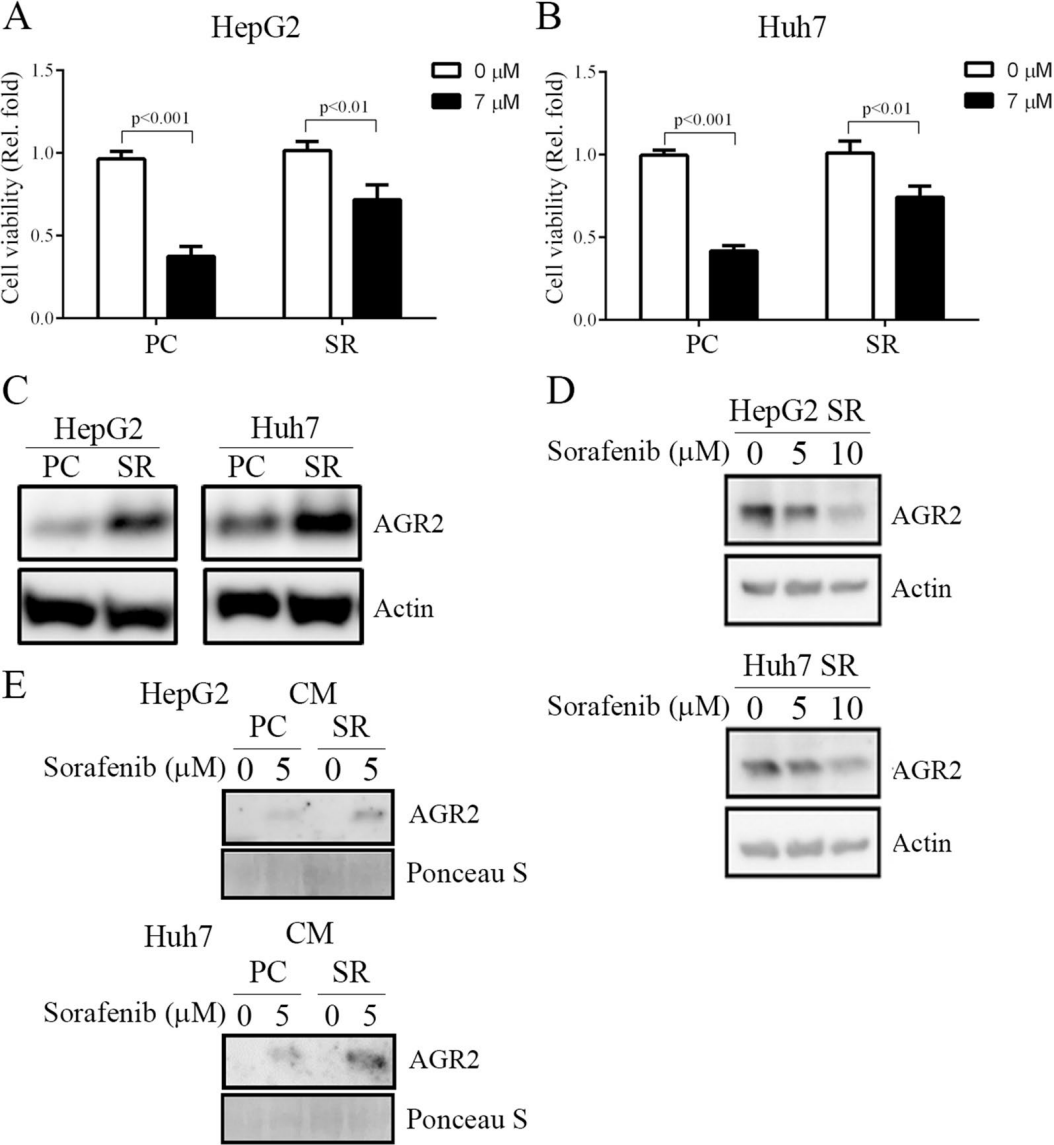
AGR2 regulation in sorafenib-sensitive and sorafenib-resistant cells. **A**–**E** Cell viability **A**, **B** and AGR2 regulation **C**–**E** in both cell lysate **C**, **D** and CM (CM, E) from parental cells (PC) and sorafenib-resistant (SR) cells treated with sorafenib. Sorafenib-resistant cells show higher cell viability, and AGR2 is highly expressed in sorafenib-resistant cells compared to sorafenib-sensitive cells. 0: untreatment; 5, 10: sorafenib treatment

To determine whether AGR2 plays diverse roles in resistant sublines compared to sorafenib-sensitive HCC cells, HepG2 sorafenib-resistant (HepG2-SR) and Huh7 sorafenib-resistant (Huh7-SR) cells were established (Fig. 5A, B). We applied 7 µM sorafenib to the culture medium to generate sorafenib-resistant cell lines for the following experiments. Using MTT assay, we found that cell viability was decreased by approximately 50% after sorafenib treatment (7 μM) in parental HepG2 and Huh7 cells (indicated as PCs); however, sorafenib only reduced cell viability by 10–20% in resistant cells (indicated as SR cells) (Fig. 5A, B), indicating that these SR cell lines are protected against sorafenib challenge. We found that AGR2 was related to resistance and upregulated by sorafenib in the GSE94550 dataset (Fig. 1A, B). Therefore, we verified whether this regulation was observed in our sorafenib-resistant cells. As expected, AGR2 was highly expressed intracellularly in sorafenib-resistant HepG2 and Huh7 cells compared with parental cells (lane 2 vs 1; Fig. 5C; Additional file 7: figure S7A). We also found that sorafenib reduced intracellular AGR2 levels in HepG2-SR and Huh7-SR cells (lane 3 vs 1; Fig. 5D; Additional file 7: figure S7B), and the tendency was similar to that of sorafenib-regulated AGR2 in HepG2-PCs and Huh7-PCs (Fig. 2M–P). Moreover, we found that sorafenib can induce AGR2 secretion in CM in HepG2-SR and Huh7-SR cells compared with controls. The effect was stronger in resistant cells than in parental cells (lane 4 vs 3; Fig. 5E; Additional file 7: figure S7C). Collectively, these results indicate that AGR2 induction was more robust in both the cell lysate and CM of sorafenib-resistant cells than in parental cells in the presence and absence of sorafenib. This finding might explain why SR cells are protected against sorafenib.

### Sorafenib-resistant cells modulate ER stress and reduce cell apoptosis

**Fig. 6:**
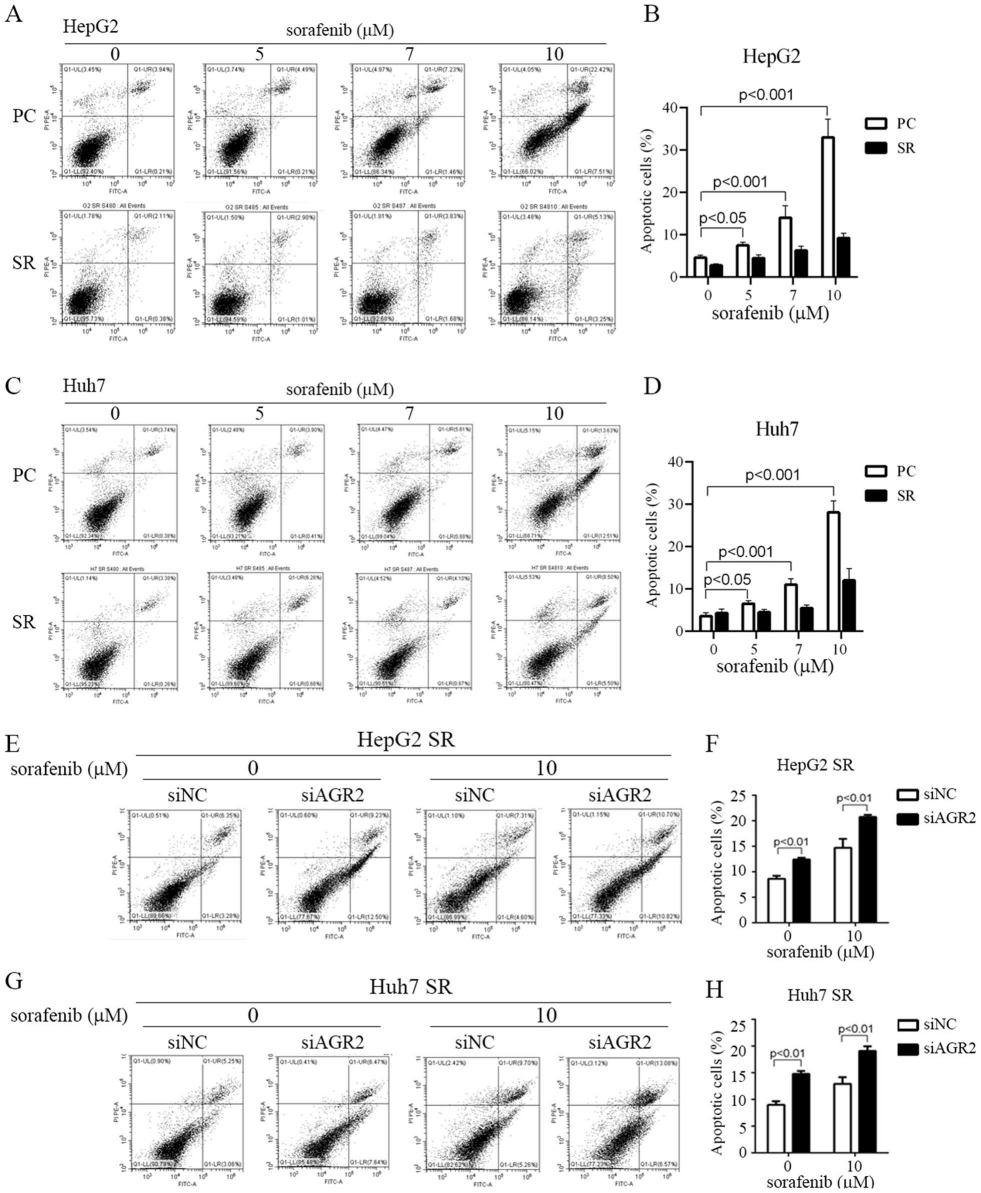
AGR2 is involved in HCC resistance to sorafenib. **A**–**D** Apoptosis assay performed after stimulation of both HepG2 and Huh7 parental cell s (PC) and sorafenib-resistant (SR) cells with sorafenib at various concentrations (0–10 μM). The apoptosis rate was lower in sorafenib-resistant cells (indicated as SR cells) than in parental cells (indicated as PCs). Sorafenib-resistant cells have higher protection and resistance to sorafenib-induced toxicity. **E**–**H** Apoptosis assay performed in HepG2 SR and Huh7 SR cells with AGR2 silencing in the presence of sorafenib (10 μM). More apoptotic of HepG2 and Huh7 cells were observed in the AGR2-silenced SR group than in the vector control group. Sorafenib-induced apoptosis is more obvious with AGR2 silencing. siNC: negative control siRNA, siRNA vector only; siAGR2: AGR2 siRNA

To determine whether sorafenib-resistant cells can resist the effect of sorafenib-induced cell apoptosis, flow cytometry analysis was utilized to demonstrate that cell apoptosis was induced after stimulation with various doses of sorafenib for 24 h in HepG2 (Fig. 6A, B) and Huh7 (Fig. 6C, D) parental cells. The ratio of cells undergoing sorafenib-induced apoptosis reached approximately 30% among parental cells; however, the effect was not observed in sorafenib-resistant cells (Fig. 6A–D). Based on this evidence, we suggest that these resistant cells are protected against sorafenib toxicity, which prolongs cancer cell viability. Furthermore, we analyzed whether AGR2 plays a vital role in inducing HCC resistance to sorafenib. We silenced AGR2 in HepG2 SR and Huh7 SR cells and performed an apoptosis assay. The results indicated that sorafenib can induce cell apoptosis, but the effect was more robust after AGR2 knockdown (Fig. 6E–H). According to these findings, we speculate that AGR2 plays a critical role in inducing sorafenib resistance in HCC.

**Fig. 7:**
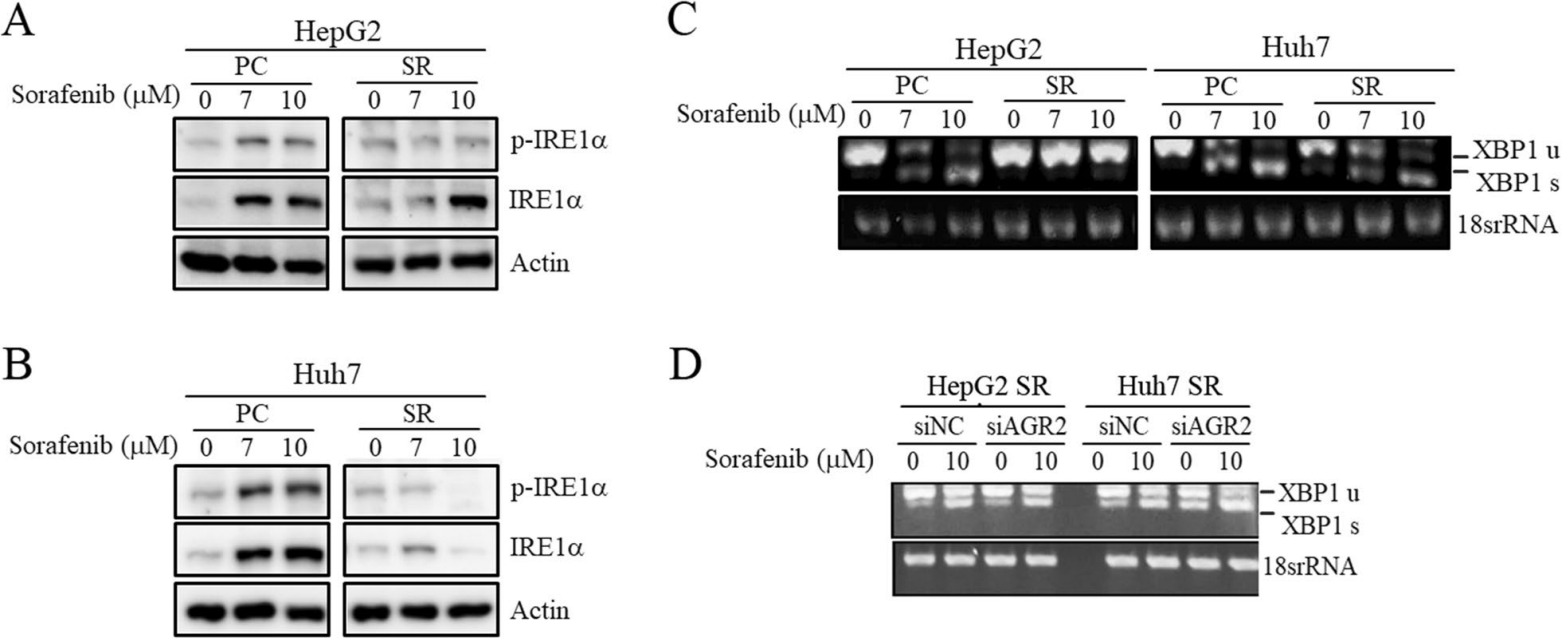
AGR2 is involved in the sorafenib-regulated IRE1α-XBP1 cascade. **A**, **B**, **C**, **D** The RNA **C**, **D** and protein **A**, **B** levels of the ER stress-related molecules p-IRE1α, IRE1α **A**, **B**, and XBP1 (u and s, **C**, **D**) were examined via Western blotting **A**, **B** and RT–PCR **C**, **D** after AGR2 silencing **D** and treatment with sorafenib at concentrations ranging from 0 to 10 μM in both parental (PC) and sorafenib-resistant (SR) HepG2 (**A**, (**C**, **D**, left)) and Huh7 (**B**, (**C**, **D**, right)) cells. Sorafenib-induced p-IRE-1α, IRE-1α regulation and XBP-1 splicing in sorafenib-sensitive cells are attenuated in sorafenib-resistant cells. u: unspliced; s: spliced; siNC: negative control siRNA, siRNA vector only; siAGR2: AGR2 siRNA. 0: untreatment; 7, 10: sorafenib treatment

We found that the ER stress-related molecule p-IRE1α was induced by sorafenib treatment; subsequently, we determined whether p-IRE1α regulation occurred in sorafenib-resistant cells. Western blot analysis revealed that the levels of p-IRE1α were decreased after sorafenib stimulation in SR HepG2 and Huh7 cells and that the effect was inconsistent in PCs (lane 2 vs 1; Fig. 7A, B; Additional file 8: Figure S8A). Moreover, according to RT–PCR results, we found that the sorafenib-induced changes in the levels of XBP1 s, a downstream factor of IRE1α, were abolished or attenuated in HepG2-SR and Huh7-SR cells and that this was not observed in parental HepG2 and Huh7 cells (lane 6 vs 3; Fig. 7C; Additional file 8: Figure S8B). Furthermore, we evaluated whether the effect was mediated by AGR2 in sorafenib-resistant cells. The data indicated that XBP1 s expression was induced after silencing AGR2 expression and was further elevated after stimulation with sorafenib (lane 4 vs 2; Fig. 7D; Additional file 8: Figure S8C). We analyzed the cleaved (C) caspase3 and AGR2 levels in tumors from nude mice subcutaneously injected with Huh7 PCs and SR cell lines, both treated with sorafenib (defined in the figure as sora PC and sora SR, respectively). Immunohistochemistry results indicated that AGR2 was more highly expressed in sora-treated SR cells than in sora-treated PC cells; in contrast, c-casp3 was slightly more highly expressed in sora-treated PC cells than in sora-treated SR cells (Additional file 9: Figure S9). This suggests that AGR2 is more essential under cell stress conditions. Collectively, the contradictory evidence in sorafenib-sensitive and sorafenib-resistant cells may indicate that cells can resist sorafenib toxicity by modulating ER stress-related molecules to decrease cellular ER stress.

**Fig. 8:**
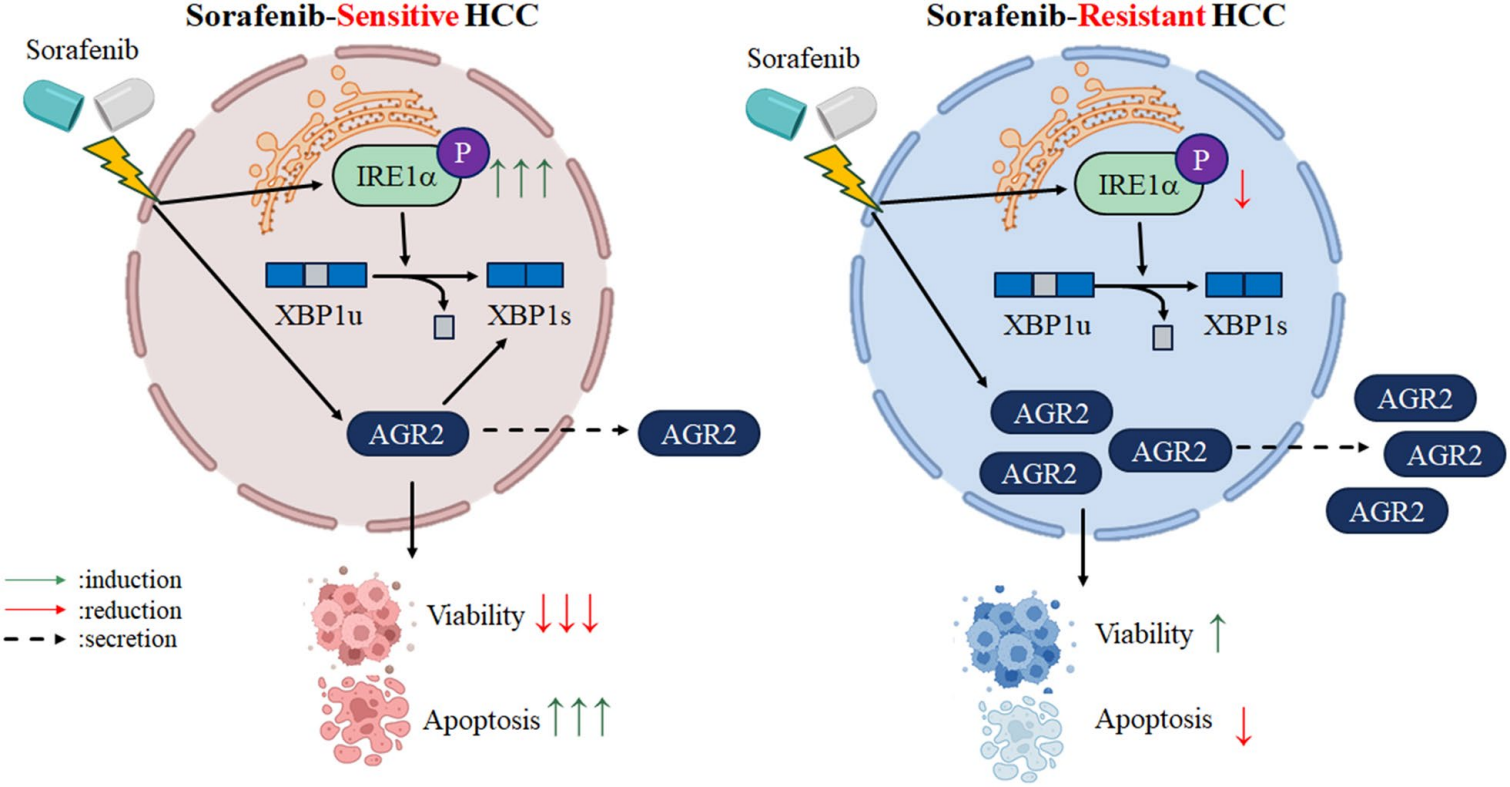
Proposed model of the roles of AGR2 in sorafenib-sensitive and sorafenib-resistant HCC. AGR2 plays different roles in sorafenib-sensitive (PC) and sorafenib-resistant (SR) cells. Sorafenib decreases AGR2 expression in the cell lysate and in turn induces secretion into CM. Sorafenib reduces cell viability and induces cell apoptosis. However, AGR2 is highly expressed in sorafenib-resistant cells, and the induction of AGR2 expression in CM was more robust in sorafenib-resistant cells than in parental cells, which in turn induces cell survival and suppresses cell apoptosis

Based on the evidence, we propose that sorafenib reduces cell viability and induces cell apoptosis via downregulation of AGR2 in the cell lysate and increased secretion in CM, which induces ER stress via upregulation of p-IRE1α and spliced XBP1 in HCC. However, the phenomenon of sorafenib-induced apoptosis was abolished in sorafenib-resistant cells, and this effect may occur through increased AGR2 expression in cell lysates and downregulation of p-IRE1α and spliced XBP1. Overall, AGR2 might modulate ER stress to protect cells from sorafenib toxicity and extend cell survival (Fig. 8).

## Discussion

In the present study, we found that AGR2 is significantly correlated with OS, RFS, and various clinical parameters, including AFP, ALT, the predicted risk metastasis signature score, tumor size, and pathological stage. The 160 clinical HCC specimens were divided into high and low groups according to the AGR2 level determined by qRT‒PCR, with a cut-off at 10.8 (39-ΔCt). The 120 patients with AGR2 levels ≥ 10.8 (39-△Ct) presented worse recurrence-free-survival (P = 0.045) (Table 5, Fig. 1K) and overall survival rates (P = 0.016) (Table 6, Fig. 1J) than the 40 patients with AGR2 levels ≤ 10.8 (39-△Ct). However, the AGR2 levels in HCC patients before and after sorafenib treatment remain unknown. Furthermore, we intend to investigate the AGR2 levels in patients treated with and without sorafenib to analyze the ratio of AGR2 levels under both conditions, which might provide a precise ratio for chemotherapy outcomes and aid in determining the clinical prognosis of patients with HCC. We utilized sorafenib-sensitive and sorafenib-resistant cells to determine the roles of AGR2 in HCC progression and drug resistance. We suggest that AGR2 plays diverse roles and has different mechanisms through which it influences HCC progression and sorafenib resistance in these two models. Functionally, AGR2 knockdown reduces cell viability and induces cell apoptosis with sorafenib treatment in parental HCC cell lines. However, the phenomenon of sorafenib-induced cell apoptosis in sorafenib-sensitive cells is almost abolished in sorafenib-resistant cells. Mechanistically, sorafenib modulates AGR2 through posttranslational modification instead of transcriptional regulation and activates the IRE1α-XBP1 cascade to induce death in parental cells, but this effect is not observed in sorafenib-resistant cells. This is the first report to uncover the role of AGR2 in sorafenib-resistant HCC and to explain how AGR2 induces HCC resistance to sorafenib and reduces cell apoptosis.

The regulation of ER stress-related molecules by sorafenib is also diverse in these two models. Collectively, our preliminary data highlight a novel regulatory mechanism of AGR2 that may serve as a critical determinant of cancer progression and drug resistance in HCC. Cancer cells often initiate ER stress via misfolded protein accumulation in the ER due to protein overexpression, nutrient deprivation or hypoxia. Cells can activate UPR signaling to trigger ER homeostasis and prolong cell survival [35]. AGR2 is an ER-resident protein that catalyzes thiol-disulfide interchange and protein folding reactions [11, 12]. Our results showed that dysregulated AGR2 expression and ER stress-related molecules appear in sorafenib-sensitive and sorafenib-resistant cells. Decreased AGR2 and increased p-IRE1α and spliced XBP1 levels induced by sorafenib were observed in the cell lysate of sorafenib-treated sensitive cells; in contrast, increased AGR2 in CM and decreased p-IRE1α and spliced XBP1 expression induced by sorafenib appeared in sorafenib-treated resistant cells. Blazanin and colleagues reported that v-Ha-Ras, an oncogenic protein, can induce ER stress partially through upregulation of both the mRNA and protein levels of total IRE1α and that phosphorylated IRE1α was also induced by ER stress [36, 37]. Moreover, keratinocytes treated with the ER stress inhibitor 4-phenyl butyric acid (4-PBA) exhibited increased levels of both total IRE1α and phosphorylated IRE1α [36]. Based on the evidence, we suggest that the mRNA and protein levels of IRE1α will be upregulated, followed by upregulation of phosphorylated IRE1α, upon ER stress. Our findings of sorafenib-induced ER stress via upregulation of both total and phosphorylated IRE1α are similar to the findings of other groups. According to these diverse effects, we speculate that the ER stress level differed in these two cell models. Sorafenib induced higher ER stress in parental HCC cells; however, sorafenib-induced ER stress was attenuated in sorafenib-resistant cells, indicating that ER stress might be a critical factor in determining whether cells can resist sorafenib. Therefore, we suggest that ER stress homeostasis is a therapeutic target to influence the status of sorafenib resistance in HCC.

In the present study, cell viability and cell apoptosis were altered in Hep3B, HepG2 and Huh7 parental cells upon sorafenib treatment after silencing AGR2. AGR2 contributes to ER homeostasis via UPR signaling, including the IRE1α-XBP1 cascade [29]. In the present study, we found that silencing AGR2 in the presence or absence of sorafenib increased spliced XBP1 levels in both sorafenib-sensitive and sorafenib-resistant cells. We further found that exogenous recombinant AGR2 can reduce sorafenib-induced XBP1 splicing. However, whether XBP1 is an upstream effector that modulates AGR2 expression during HCC progression and resistance to sorafenib is unclear. Some ER stress inhibitors, including tauroursodeoxycholic acid (TUDCA, 100 μM, [38]) and the IRE1α endonuclease inhibitor MKC-3946 (10 μM, [39]), can be used to inhibit ER stress to further delineate the relationship between AGR2 and ER stress in both sorafenib-sensitive and sorafenib-resistant cells. Collectively, our findings provide a new mechanism by which AGR2 might act as an upstream factor of XBP1 to modulate ER homeostasis and influence the cell death or survival status in sorafenib-sensitive and sorafenib-resistant HCC.

We preliminarily identified the functions and regulatory mechanisms of AGR2 in the response to sorafenib. AGR2 has been demonstrated to be upregulated upon ER stress, and ER stress-related molecules, such as PERK, IRE1 and ATF6, are dysregulated in many cancer types [16]. Herein, we demonstrated that AGR2 can modulate the IRE1α-XBP1 cascade to modulate ER homeostasis, switching HCC from the sorafenib-sensitive to the sorafenib-resistant type. However, in this study, other ER stress-related signaling molecules were shown to be affected by sorafenib; the levels of total ATF6, cleaved ATF6 and p-PERK were regulated by sorafenib, as shown by Western blotting, but the regulatory effects of sorafenib were slightly weaker than those of IRE1α. Hence, we speculate that ATF6 and PERK may constitute another potential pathway regulated by AGR2 that influences cancer cell progression and resistance to sorafenib. We used the cBioPortal software for Cancer Genomics developed by the Memorial Sloan Kettering Cancer Center (MSKCC) [40–42]. AGR2 expression has been demonstrated to be negatively correlated with ATF6 expression (Spearman: − 0.19, p value: 2.15e-4) based on the TCGA, Filehorse microarray dataset. Moreover, based on the TCGA, Pancancer microarray dataset, a negative correlation between AGR2 and ATF6 expression was also reported (Spearman: − 0.2, p value: 9.174e-5). Based on the online published microarray datasets, we suggest that AGR2 has a significant correlation with ER stress in HCC. According to the cBioPortal software information, several correlations between AGR2 and ER stress-related factors have been demonstrated in numerous cancer types, such as HCC and lung, breast and pancreatic cancers. The AGR2 mRNA levels were negatively correlated with XBP1 protein levels in HCC (Spearman: − 0.169, p value: 0.02) in the TCGA, Pancancer microarray dataset and in lung squamous cell carcinoma (Spearman: − 0.16, p value: 3.65e-3). Furthermore, a negative correlation between AGR2 and ATF6 was also reported in TCGA, lung squamous cell carcinoma (Spearman: − 0.104, p value: 0.02) and TCGA, pancreatic (Spearman: − 0.32, p value: 5.015e-3) cancer. Based on analyses of these published microarray datasets, AGR2 is highly correlated with ER stress-related molecules, such as XBP-1 and ATF6, and these results were similar to our findings, making our study more complete. In conclusion, we suggest that the AGR2-IRE1α-XBP1 cascade is an ER-related pathway that regulates HCC progression; hence, this signaling cascade might be a potential therapeutic target for curing sorafenib-resistant HCC in the future.

Previous research has shown that intracellular AGR2 (iAGR2) can promote cancer cell growth and survival and that extracellular AGR2 (eAGR2) can be defined as a microenvironment regulator that makes cancer cells more aggressive [21, 29]. We found that AGR2 can be detected in CM from both sorafenib-sensitive and sorafenib-resistant cells, and the induction ratio with sorafenib treatment was more robust in resistant cells than in sensitive cells. However, the roles of iAGR2 and eAGR2 in the presence of sorafenib are still unclear. Fessart et al. reported that extracellular AGR2 is an extracellular matrix pro-oncogenic regulator that makes cancer cells more aggressive [21]. AGR2 has been demonstrated to have numerous domains, contributing to diverse functions in cancer cells [32]. However, whether these domains are associated with cancer progression and drug resistance in the presence of sorafenib is not clear. The functions of AGR2 domains in both sorafenib-sensitive and sorafenib-resistant cells need to be elucidated in more detail using truncated AGR2 mutants. Two truncated AGR2 forms, including deletions of amino acids (AAs) 1-20 and AAs 172-175, which can be localized to the extracellular space [32], can be utilized in the future. Using these AGR2 mutants, we will be able to determine whether the subcellular location of AGR2 plays a critical role in regulating cancer progression and sorafenib resistance.

Sorafenib has been demonstrated to inhibit numerous receptor tyrosine kinases, such as VEGFR and PDGFR [4]. Moreover, extracellular AGR2 has been shown to bind directly to VEGF to enhance tumor angiogenesis and other activities [43]. We found that AGR2 can be secreted into CM after sorafenib stimulation of sorafenib-resistant HepG2 and Huh7 cells, but this was not observed in parental cells. However, the mechanism underlying AGR2-induced resistance to sorafenib in HCC has never been elucidated. Hence, it is necessary to analyze whether recombinant AGR2 can directly interact with recombinant VEGF. Previously, several VEGFR- and PDGFR-related signaling pathways, including the RAS, RAF, MEK, ERK, PI3K/Akt and JAK-STAT pathways, have been reported to be inhibited by sorafenib [6]. However, the signaling underlying AGR2-induced resistance to sorafenib in HCC has never been elucidated. Therefore, in the future, these pathways need to be examined in sorafenib-sensitive and sorafenib-resistant cells with AGR2 silencing and AGR2 overexpression in the presence of sorafenib. Elucidation of the predictive role and molecular and cellular mechanisms of AGR2 related to sorafenib resistance can provide additional opportunities to establish complementary therapies for HCC.

## Supplementary Information

**Additional file 1: Figure S1.** The RNA (A–D) and protein (E–J) levels both cell lysate (E–H) and conditioned medium (I, J, CM) of AGR2 stimulated with sorafenib (5–10 μM) in J7 (A, E), HepG2 (B, F, I), Huh7 (C, G) and Hep3B (D, H, J) cells using RT-PCR (A–D) and Western blotting (E–J) were quantified (normalized with untreatment (0) control).

**Additional file 2: Figure S2.** The AGR2 levels with transfection of AGR2 siRNA (siAGR2) and vector control (siNC) in Hep3B (A), HepG2 (B) and Huh7 (C) cells were quantified (normalized with siNC control). NC: negative control.

**Additional file 3: Figure S3.** The protein levels of cleaved (C) ATF6, phosphate (p)-IRE1α and p-PERK with sorafenib (5-10 μM) stimulation in HepG2 (A) and Huh7 (B) cells were quantified (normalized with untreatment (0) control).

**Additional file 4: Figure S4.** J7 and Huh7 cells were stimulated with 5 and 10 μM sorafenib, followed by examination of Bip expression using Western blotting. 0: untreatment control.

**Additional file 5: Figure S5.** The spliced XBP1 (XBP1s) levels with sorafenib (5–10 μM) stimulation (A, B), transfection of AGR2 siRNA (siAGR2) and vector control (siNC) (C, D), or recombinant (r) AGR2 stimulation (E, F) in HepG2 (A, C, E) and Huh7 (B, D, F) cells were quantified. NC: negative control. (normalized with untreatment (0) control)

**Additional file 6: Figure S6.** The levels of AGR2 and unspliced (u) XBP1 and spliced (s) XBP1 were determined by RT‒PCR in 9 normal tissues and 5 HCC tissues. 18S rRNA was used as an internal control.

**Additional file 7: Figure S7.** The protein levels of AGR2 both cell lysate (A, B) and conditioned medium (C, CM) in parental cells (PC) or sorafenib-resistant (SR) HepG2 (A-C, left) and Huh7 (A-C, right) cells in the presence or absence of sorafenib (5–10 μM) detected by Western blotting were quantified. (normalized with untreatment (0) control)

**Additional file 8: Figure S8.** The protein level of phosphate (p)-IRE1α (A) and RNA level of XBP1 (XBP1s) (B-C) detected by Western blotting (A) and RT-PCR (B–C) with sorafenib (7–10 μM) stimulation (A, B), transfection of AGR2 siRNA (siAGR2) and vector control (siNC) (C) in parental cells (PC) and sorafenib-resistant (SR) HepG2 (A-C, left) and Huh7 (A–C, right) cells were quantified.

**Additional file 9: Figure S9.** The levels of AGR2 and cleaved (c) caspase3 (CASP3) were determined by immunohistochemistry in vivo in nude mice injected with sorafenib (sora)-treated Huh7 parental cells (PCs) and sora-treated Huh7 resistant (SR) cells.

## Data Availability

Not applicable.
