# Supplementary figures and images for: Anterior gradient 2 induces resistance to sorafenib via endoplasmic reticulum stress regulation in hepatocellular carcinoma
Source: Cancer Cell Int. 2023 Mar 10;23:42. doi: 10.1186/s12935-023-02879-w (PMC9999520; doi:10.1186/s12935-023-02879-w)

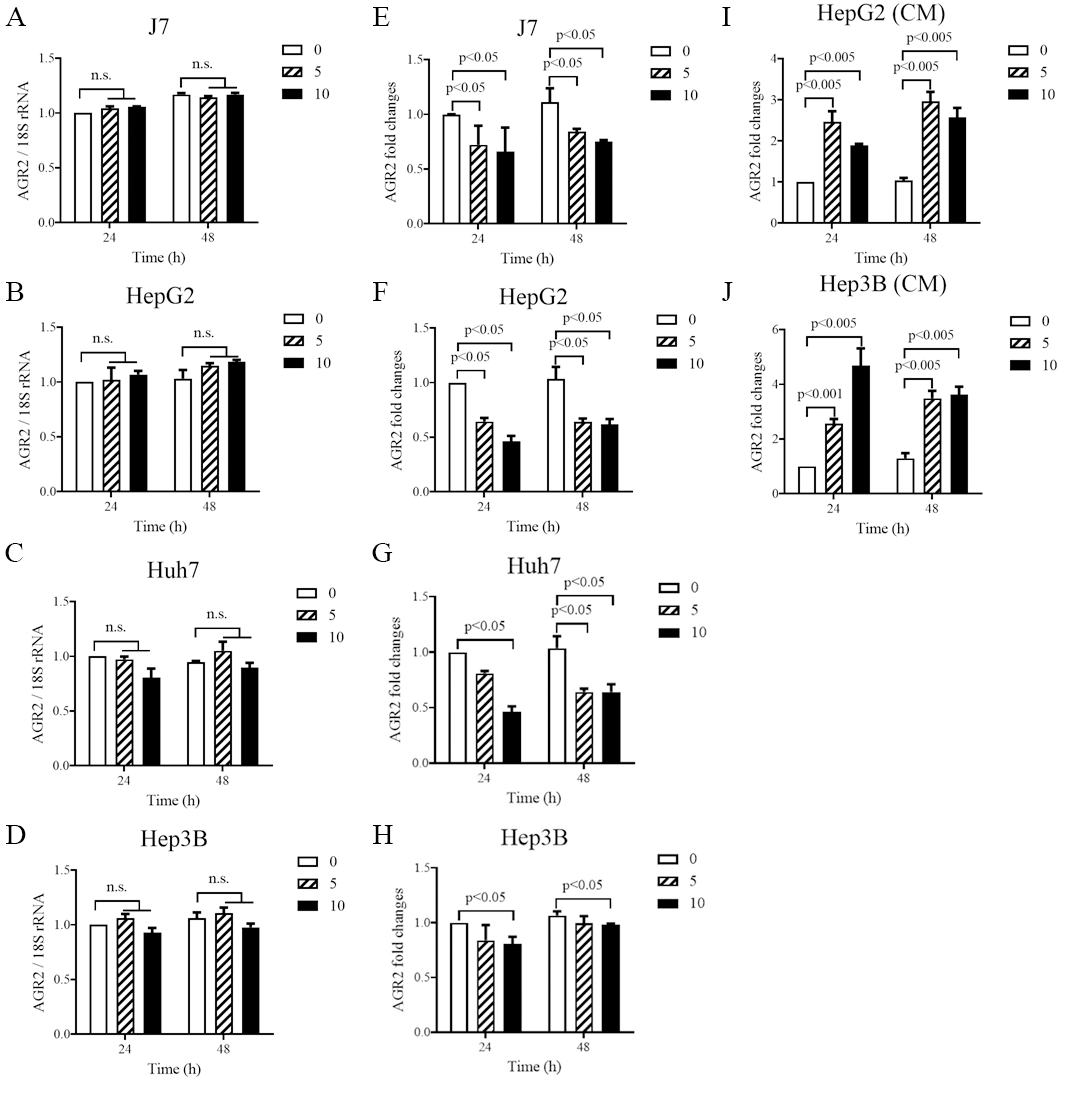

Supplement: Supplementary file 1 — Additional file 1: Figure S1. The RNA (A–D) and protein (E–J) levels both cell lysate (E–H) and conditioned medium (I, J, CM) of AGR2 stimulated with sorafenib (5–10 μM) in J7 (A, E), HepG2 (B, F, I), Huh7 (C, G) and Hep3B (D, H, J) cells using RT-PCR (A–D) and Western blotting (E–J) were quantified (normalized with untreatment (0) control). [file 12935_2023_2879_MOESM1_ESM.tif]

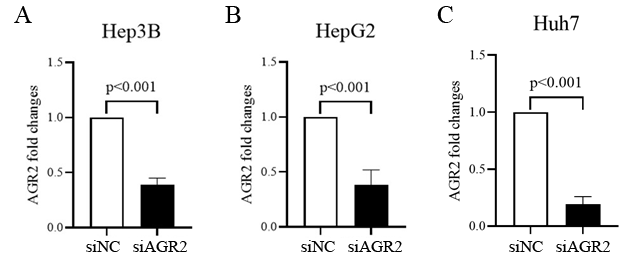

Supplement: Supplementary file 2 — Additional file 2: Figure S2. The AGR2 levels with transfection of AGR2 siRNA (siAGR2) and vector control (siNC) in Hep3B (A), HepG2 (B) and Huh7 (C) cells were quantified (normalized with siNC control). NC: negative control. [file 12935_2023_2879_MOESM2_ESM.tif]

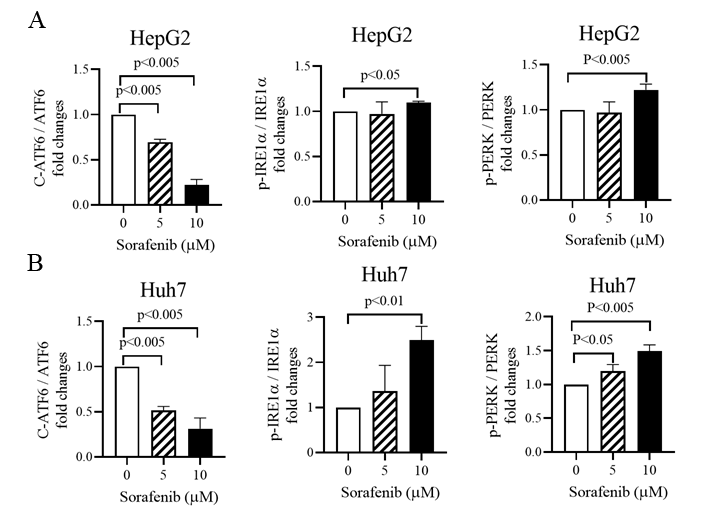

Supplement: Supplementary file 3 — Additional file 3: Figure S3. The protein levels of cleaved (C) ATF6, phosphate (p)-IRE1α and p-PERK with sorafenib (5-10 μM) stimulation in HepG2 (A) and Huh7 (B) cells were quantified (normalized with untreatment (0) control). [file 12935_2023_2879_MOESM3_ESM.tif]

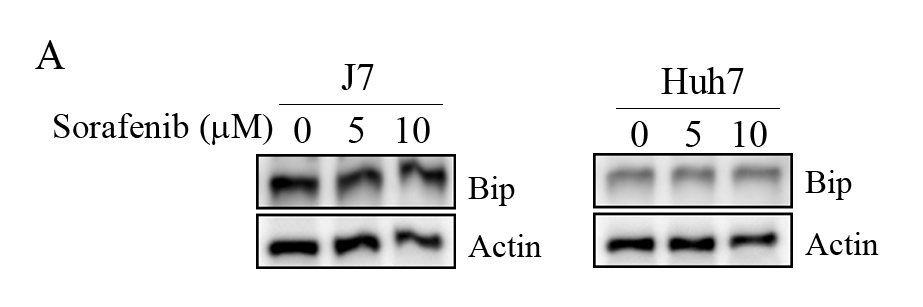

Supplement: Supplementary file 4 — Additional file 4: Figure S4. J7 and Huh7 cells were stimulated with 5 and 10 μM sorafenib, followed by examination of Bip expression using Western blotting. 0: untreatment control. [file 12935_2023_2879_MOESM4_ESM.tif]

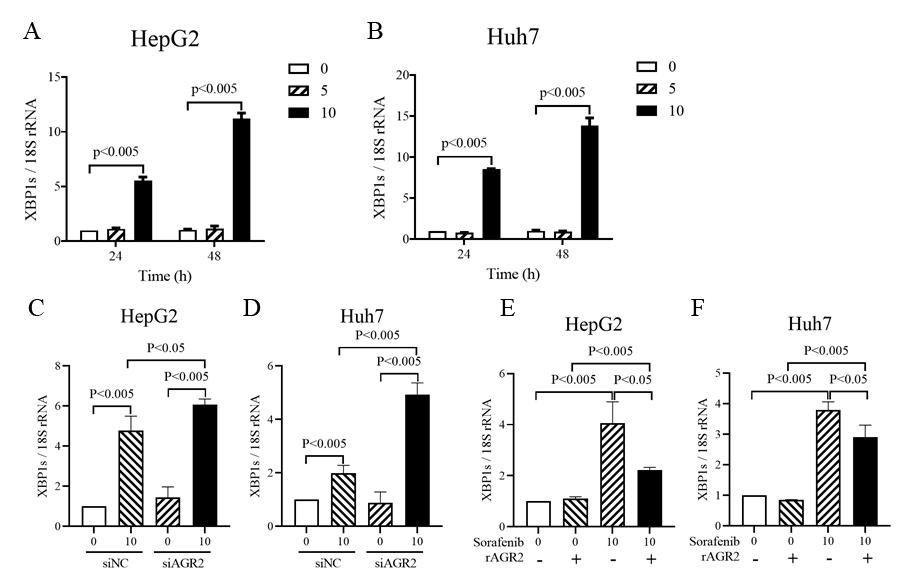

Supplement: Supplementary file 5 — Additional file 5: Figure S5. The spliced XBP1 (XBP1s) levels with sorafenib (5–10 μM) stimulation (A, B), transfection of AGR2 siRNA (siAGR2) and vector control (siNC) (C, D), or recombinant (r) AGR2 stimulation (E, F) in HepG2 (A, C, E) and Huh7 (B, D, F) cells were quantified. NC: negative control. (normalized with untreatment (0) control) [file 12935_2023_2879_MOESM5_ESM.tif]

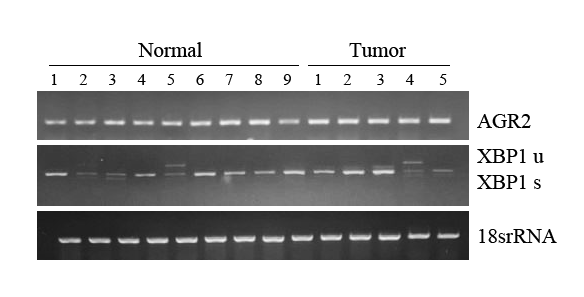

Supplement: Supplementary file 6 — Additional file 6: Figure S6. The levels of AGR2 and unspliced (u) XBP1 and spliced (s) XBP1 were determined by RT‒PCR in 9 normal tissues and 5 HCC tissues. 18S rRNA was used as an internal control. [file 12935_2023_2879_MOESM6_ESM.tif]

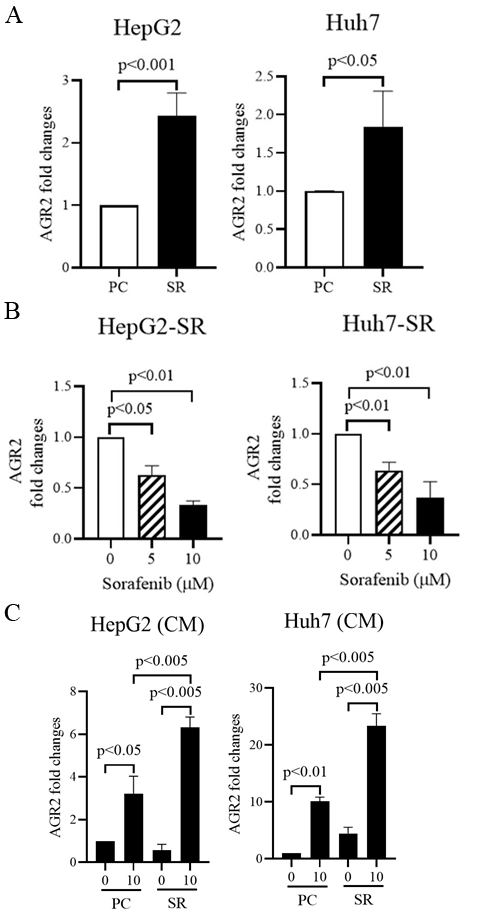

Supplement: Supplementary file 7 — Additional file 7: Figure S7. The protein levels of AGR2 both cell lysate (A, B) and conditioned medium (C, CM) in parental cells (PC) or sorafenib-resistant (SR) HepG2 (A-C, left) and Huh7 (A-C, right) cells in the presence or absence of sorafenib (5–10 μM) detected by Western blotting were quantified. (normalized with untreatment (0) control) [file 12935_2023_2879_MOESM7_ESM.tif]

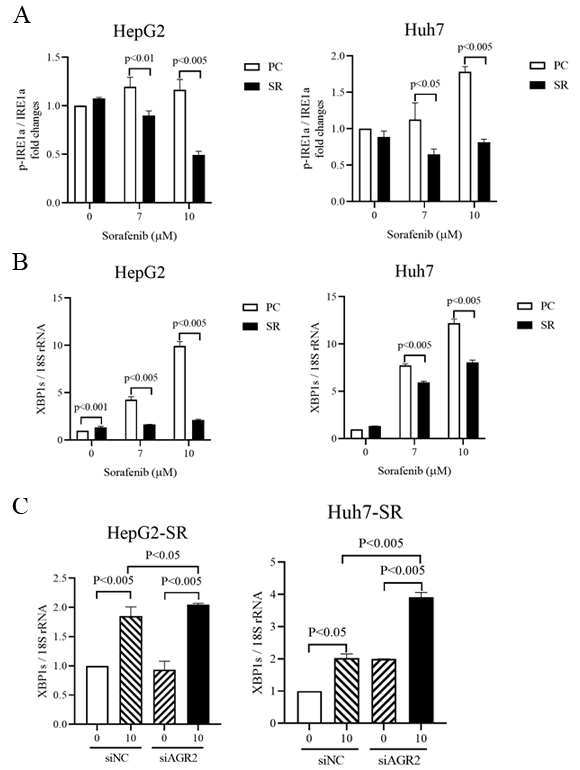

Supplement: Supplementary file 8 — Additional file 8: Figure S8. The protein level of phosphate (p)-IRE1α (A) and RNA level of XBP1 (XBP1s) (B-C) detected by Western blotting (A) and RT-PCR (B–C) with sorafenib (7–10 μM) stimulation (A, B), transfection of AGR2 siRNA (siAGR2) and vector control (siNC) (C) in parental cells (PC) and sorafenib-resistant (SR) HepG2 (A-C, left) and Huh7 (A–C, right) cells were quantified. [file 12935_2023_2879_MOESM8_ESM.tif]

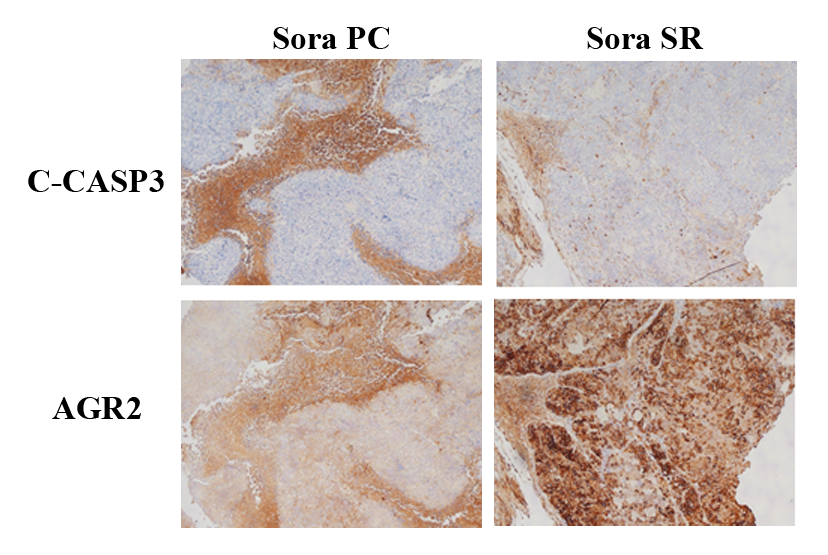

Supplement: Supplementary file 9 — Additional file 9: Figure S9. The levels of AGR2 and cleaved (c) caspase3 (CASP3) were determined by immunohistochemistry in vivo in nude mice injected with sorafenib (sora)-treated Huh7 parental cells (PCs) and sora-treated Huh7 resistant (SR) cells. [file 12935_2023_2879_MOESM9_ESM.tif]
